# AIP1 Regulates Ocular Angiogenesis Via NLRP12‐ASC‐Caspase‐8 Inflammasome‐Mediated Endothelial Pyroptosis

**DOI:** 10.1002/advs.202405834

**Published:** 2024-11-11

**Authors:** Yonghao Li, Yimeng Sun, Dasen Xie, Hui Chen, Qi Zhang, Shaochong Zhang, Feng Wen, Jing‐Song Ou, Min Zhang, Lishi Su, Xuri Li, Wei‐Ping Wen, Wei Chi

**Affiliations:** ^1^ Shenzhen Eye Hospital Shenzhen Key Laboratory of Ophthalmology Jinan University Shenzhen Guangdong 518043 China; ^2^ State Key Laboratory of Ophthalmology Zhongshan Ophthalmic Center Guangdong Provincial Key Laboratory of Ophthalmology and Visual Science Guangdong Provincial Clinical Research Center for Ocular Diseases Sun Yat‐Sen University Guangzhou Guangdong 510060 China; ^3^ Xiamen Key Laboratory of Ophthalmology Xiamen Eye Center and Eye Institute of Xiamen University Xiamen Fujian 361003 China; ^4^ Division of Cardiac Surgery Cardiovascular Diseases Institute The First Affiliated Hospital Sun Yat‐sen University Guangzhou Guangdong 510080 China; ^5^ National‐Guangdong Joint Engineering Laboratory for Diagnosis and Treatment of Vascular Diseases NHC key Laboratory of Assisted Circulation and Vascular Diseases (Sun Yat‐sen University) Key Laboratory of Assisted Circulation and Vascular Diseases Chinese Academy of Medical Sciences Guangzhou Guangdong 510080 China; ^6^ Department of Otolaryngology the Sixth Affiliated Hospital of Sun Yat‐sen University Otorhinolaryngology Institute of Sun Yat‐sen University Guangzhou Guangdong 510655 China

**Keywords:** inflammasome, pyroptosis, retina neovascularization

## Abstract

Pathological ocular angiogenesis is a significant cause of irreversible vision loss and blindness worldwide. Currently, most studies have focused on the angiogenesis factors in ocular vascular diseases, and very few endogenous anti‐angiogenic compounds have been found. Moreover, although inflammation is closely related to the predominant processes involved in angiogenesis, the mechanisms by which inflammation regulates pathological ocular angiogenesis remain obscure. In this study, a vascular endothelial cells (VECs)‐specific anti‐angiogenic factor is identified, apoptosis signal‐regulating kinase 1(ASK1)‐interacting protein‐1 (AIP1) as a key pathogenic regulator in a typical ocular angiogenesis model, oxygen‐induced retinopathy (OIR), using single‐cell RNA sequencing. It is demonstrated that AIP1 inhibited pathological angiogenesis by preventing a particular inflammatory death pathway, namely pyroptosis, in retinal VECs. The assembly of a noncanonical inflammasome is further uncovered, the NLRP12–ASC‐caspase‐8 inflammasome, which is promoted by decreased AIP1 in OIR. This inflammasome elicited gasdermin D (GSDMD)‐dependent endothelial pyroptosis, which in turn promoted the release of vascular endothelial growth factor (VEGF) and interleukin (IL)‐1β. Suppression of NLRP12–CASP8–GSDMD axis and AIP1 upregulation reduced VEGF signaling, limiting new vessel formation. These findings reveal a previously uncharacterized inflammatory angiogenic process involving VECs pyroptosis‐inducing retinal neovascularization, paving the way for promising therapeutic avenues targeting angiogenesis via AIP1 or pyroptosis.

## Graphical abstract

Pathological ocular angiogenesis leads to vision loss worldwide. This study identifies AIP1 as a key anti‐angiogenic factor in retinal vascular endothelial cells, inhibiting pathological angiogenesis by preventing GSDMD‐mediated pyroptosis via the NLRP12‐CASP8‐ASC inflammasome, offering potential new therapies for ocular angiogenesis.

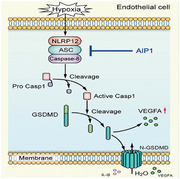

## 1 Introduction

Pathologic angiogenesis is one of the leading causes of blindness in many eye diseases across all age groups worldwide, such as retinopathy of prematurity (ROP), diabetic retinopathy (DR), and retinal vein occlusion (RVO), all characterized by an uncontrolled retinal vessel growth due to tissue hypoxia and nutrient deprivation. Pathologic retinal angiogenesis is a chronic and progressive process, typically initiated by ischemia or hypoxia due to vessel loss, which subsequently enhances the production of pro‐angiogenic vascular endothelial growth factor (VEGF), leading to excessive vessel formation that interferes with retinal light detection.^[^ ^1^ ^]^ The precise mechanisms by which retinal ischemia or hypoxia induces neovascularization are poorly understood. Moreover, targeted anti‐VEGF treatments are not always effective and have raised safety concerns, such as inhibiting normal vascularization, neuron survival, low efficacy in the later phase of retinal neovascular diseases, and serious complications like an increased risk of thromboembolic events.

Robust evidence suggests that inflammation is actively involved in pathologic angiogenesis across various ocular diseases.^[^ ^2^, ^3^, ^4^ ^]^ Our previous study demonstrated the critical role of Toll‐like receptor 4 (TLR4) in neuroinflammation associated with retinal ischemia‐reperfusion injury by promoting the activation of the NLRP1/NLRP3 inflammasome, which in turn converts immature interleukin (IL)‐1β into its mature pro‐inflammatory form.^[^ ^5^ ^]^ Meanwhile, clinical practices have started incorporating anti‐inflammatory treatments into therapeutic regimens for retinal neovascularization (RNV) diseases. Biochemical factors under hypoxic conditions, such as growth factors and inflammatory factors, are key contributors to vascular endothelial cells (VECs) dysfunction.^[^ ^6^, ^7^ ^]^ In an inflammatory environment, VECs serve as active participants and inflammation regulators. Elucidating the regulatory mechanisms of VECs dysfunction is of significant scientific importance for modulating inflammatory responses and maintaining VECs function.^[^ ^8^ ^]^

Endogenous anti‐angiogenic factors could represent promising therapeutic options for treating pathologic retinal angiogenesis. In the present study, we identified one such factor, the endogenous anti‐angiogenic factor, apoptosis signal‐regulating kinase 1–interacting (ASK1‐interacting) protein‐1 (AIP1), in the VECs of oxygen‐induced retinopathy (OIR) mice using single‐cell RNA sequencing (scRNA‐seq). VECs dysfunction is the initial event and a predictive marker for RNV.^[^ ^9^, ^10^ ^]^ The urgent need to develop endogenous anti‐angiogenic factors to suppress pathological vessel growth while promoting normal vessel growth led us to investigate the potential roles and mechanisms of AIP1 in regulating VEC dysfunction under hypoxic conditions.

Under physiological conditions, angiogenesis is regulated by the balance between VEGFA and VEGFB binding to VEGF receptor 1 (VEGFR1) and VEGF receptor 2 (VEGFR2). The affinity of VEGFR1 for VEGFA is 10‐fold higher than that for VEGFR2,^[^ ^11^ ^]^ but VEGFA binding to VEGFR1 does not appear to activate downstream angiogenic effectors, indicating that VEGFR1 acts as a decoy receptor, limiting angiogenesis through VEGFA scavenging.^[^ ^12^ ^]^ Alternatively, VEGFB binds exclusively to VEGFR1, and VEGFB overexpression accelerates new vessel formation by competitively occupying VEGFR1 sites, thereby increasing VEGFA–VEGFR2 signaling.^[^ ^13^ ^]^ Additionally, AIP1 has been suggested to interact with the VEGFR2 complex to inhibit VEGFA–VEGFR2 signaling.^[^ ^14^ ^]^ However, the mechanisms regulating AIP1 expression and its effects on VEGF signaling are still unclear.

In addition to VEGF signaling, AIP1 has been shown to suppress TLR4‐MyD88 inflammatory signaling in lipopolysaccharide (LPS)‐stimulated VECs.^[^ ^15^, ^16^, ^17^ ^]^ These findings have spurred intense efforts to develop direct anti‐inflammatory treatments for pathogenic angiogenesis, but currently, there are no effective agents in clinical practice. Given the pivotal role of TLR4 in neuroinflammation, we hypothesized that AIP1 may regulate inflammatory angiogenesis and VEGF signaling in the early stage of the disease, thus providing a potential therapeutic target. IL‐1β has also been implicated in the induction of VEGFA production, thereby serving as a molecular link between inflammation and angiogenesis.^[^ ^18^, ^19^, ^20^ ^]^ Furthermore, the inflammasome also drives inflammation‐associated cell death known as pyroptosis through cleavage of the protein gasdermin D (GSDMD), leading to the release of a pore‐forming N‐terminal product and osmotic cell lysis.^[^ ^21^ ^]^ Despite the known contribution of inflammatory signaling to pathogenic retinal angiogenesis, there is a lack of studies on the potential roles and underlying mechanisms of pyroptosis in pathologic angiogenesis. The NLRP3 inflammasome has been proposed to exert dual functions in age‐related macular degeneration (AMD), with activation associated with both drusen in dry AMD‐like injury and the prevention of neovascularization in wet AMD.^[^ ^22^ ^]^ Lukens, Udden, and our group also reported that the newly identified inflammasome NLRP12 acts as a critical mediator of autoinflammatory disease, tumor progression, and pathogen infection.^[^ ^23^, ^24^, ^25^ ^]^ However, neither its involvement in pyroptosis nor regulation by AIP1 has been examined.

In this study, we utilized scRNA‐seq, in vitro hypoxia model, and a murine OIR model to identify a novel VECs pyroptosis pathway mediating inflammatory angiogenesis, as well as a critical protective role for AIP1, which serves as a dual‐target for anti‐inflammatory and anti‐angiogenic strategies in RNV diseases.

## 2 Materials and Methods

### 2.1 Establishment of a Murine OIR Model

AIP1^−/−^ mice were generated by the Model Animal Research Center at Nanjing University (Nanjing, China). GSDMD^−/−^, NLRP12^−/−^, and CASP8^+/−^ mice were generated by the GemPharmatech (Nanjing, China). The strains were bred as heterozygotes and genotyped by PCR and sequencing. Wild‐type (WT) littermates were used as controls. Each pregnant female was placed in a separate cage and was observed daily to record the day of birth (postnatal day (P)0). Body weights of pups from different strains were recorded at P7 (**Table** **1**). Then, neonatal mice and their nursing mothers were exposed to 75% oxygen for 5 consecutive days, followed by another 5 consecutive days of normoxic exposure, and sacrificed at P17. All procedures involving animals were conducted strictly by the Association for Research in Vision and Ophthalmology (ARVO) Statement for the Use of Animals in Ophthalmic and Vision Research. All animal experiments were formally reviewed and approved by the Animal Care and Ethics Committee of the Zhongshan Ophthalmic Center (Approval number: O2021014). All efforts were made to ensure the welfare and alleviate the suffering of animals.

**Table 1:** Body weights of different mouse strains on day 7 after birth.

Mouse strains	WT	GSDMD KO	NLRP12 KO	CASP8 ^+/−^	AIP1 KO
Body weights(g)	3.113 ± 0.1848	3.108 ± 0.182 ^N.S.^	3.049 ± 0.1737 ^N.S.^	3.095 ± 0.1846 ^N.S.^	3.049 ± 0.1564 ^N.S.^

Data are presented as the mean ± SD (n = 8). N.S., not significant. ^*^ *p* < 0.05.

### 2.2 Cell Isolation for scRNA‐seq

Retinas were extracted from OIR model mice and normal postnatal day P17 sex strain mouse pups and incubated in dissociation buffer containing 1 mg mL^−1^ hyaluronidase (Sigma, H3506) and 20 000U mL^−1^ DNase I (Sigma, DN25) for 15 min at 37 °C. Enzymatic digestion was quenched by the addition of Dulbecco's Modified Eagle Medium (DMEM, Gibco) supplemented with 10% fetal calf serum (FCS, 10270106, Gibco). Digested retinas were triturated in 1 mg mL^−1^ hyaluronidase into a single cell suspension and were then resuspended at ≈900 000 mL^−1^ in phosphate‐buffered saline plus triton X (PBST) containing 1% FCS.

### 2.3 10X Genomics scRNA‐seq

A 15‐µL volume of the single‐cell suspension prepared as described (≈8000–9000 cells) was loaded into one channel of the ChromiumTM Single Cell B Chip (10X Genomics, 1000073). The Chromium Single Cell 3’Library & Gel Bead Kit v3 (10X Genomics, 1000075) was used for single‐cell barcoding, cDNA synthesis, and library preparation following the manufacturer's instructions (Single Cell 3’ Reagent Kits User Guide Version 3). Libraries were sequenced (150‐bp paired‐end reads) using an Illumina NovaSeq 6000 system.

Demultiplexing, alignment to the GRCh38 reference, and quantification of sequencing reads were performed for each sample using Cell Ranger (Version 3.0.2) with the default parameters. The filtered gene‐barcode matrices for single cells were analyzed using Seurat (V3) following the tutorial at https://satijalab.org/seurat/v3.2/integration.html. Samples with more than 20% mitochondrial genes and fewer than 500 or greater than 5000 detected genes were filtered out. Both Control and OIR datasets were collected from three biological replicates. Each sample contained different distributions of major cell types depending on the enrichment method and individual variation. To reduce this batch effect, we integrated two datasets using the canonical correlation analysis (CCA) included in the Seurat packages. After PCA to reduce dimensionality and build k‐nearest neighbor graphs (k = 20) of the cells based on Euclidean distance in 15‐D PC space, the main cell cluster was identified using the Louvain‐Jaccard graph‐based method. Filtered cells were classified using the FindClusters function in Seurat with clustering parameter resolution set to 0.5. Next, the RunUMAP function in Seurat was used to reduce high‐dimensional data into two dimensions (2D) for visualization. Last, marker genes for each cluster were identified and matched them with well‐known classes. Wilcoxon rank‐sum test is employed to compare gene expression levels across different cell populations, identifying genes with significant differences in expression. Visualization of these differences is facilitated through volcano plots. Utilizing Seurat, violin plots are generated based on previously defined cell populations, illustrating the distribution of target gene expression levels within each cluster. The gene set enrichment analysis (GSEA) software (version 3.0, available at http://www.broadinstitute.org/gsea) was utilized to conduct GSEA based on the single gene AIP1. Visualization of the enriched pathways and functional gene sets associated with variations in AIP1 expression was achieved through the use of ridge plots.

### 2.4 Reagents and Antibodies

The following antibodies were used for western blotting (WB), co‐immunoprecipitation (co‐IP), and immunofluorescence (IF) staining as listed in **Table** **2**.

**Table 2:** The antibodies used in the article.

Antibody	Supplier	Item	Dilution ratio
anti‐VEGFA	ABclonal	#A5708	1:500
anti‐VEGFR2	ABclonal	#A5609	1:500
anti‐NLRP12	ABclonal	#A6671	1:500
anti‐AIP1	ABclonal	#A9572	1:500 for WB
anti‐AIP1	Santa Cruz	#sc‐365921	1:100 for co‐IP
anti‐CASP8	Cell Signaling Technology	#8592S	1:100 for IF
anti‐CASP8	Cell Signaling Technology	#9746S	1:500 for WB 1:100 for co‐IP
Anti‐GSDMD	Abcam	#ab210070	1:1000 for WB
Anti‐HIF‐1α	ABclonal	#A22041	1: 1000
anti‐IL1β	ABclonal	#A1112	1:200
anti‐IL‐1β	eBioscience	#16‐7018‐81	1:200
anti‐CD31	BD	553370	
anti‐VEGFB	Immunoway	#YT4871	1:500
anti‐VEGFB	Santa Cruz	#sc‐80442	1:500
anti‐CASP1	ABclonal	#A0964	1:500
anti‐NLRP12	Abcam	#ab105409	1:100 for IF
anti‐ASC	Santa Cruz	#sc‐271054	IF 1:100 co‐IP 1:100 WB 1:500
anti‐NLRP12	Biotech	#NB100‐56157	co‐IP 1:100
anti‐β‐actin	Bioworld	#AP0060	1:1000
FITC anti‐mouse CD31 antibody	BioLegend	#102405	
VEGF PE‐conjugated antibody	R&D Systems	#IC2931P	
Isolectin B4	Invitrogen	#1868228	1:200
Lipofectamine 3000	Invitrogen	#1952383	
Evans blue	Sigma		

### 2.5 Assessment of Retinal Vascular Permeability

Retinal vascular permeability was assessed using the Evans blue leakage assay as previously described.^[^ ^26^ ^]^ Briefly, OIR and control mice were injected with Evans blue dye at 30 mg kg^−1^ on P17, and the dye was allowed to circulate for 2 h while the mice were placed on an electric blanket. Mice were then perfused with saline through the left ventricle to clear Evans blue from the bloodstream. Retinas were carefully removed, dried with absorbent paper, weighed, and incubated in 150 µL formamide for 18 h at 70 °C. The formamide extract was centrifuged at 14 000 g for 30 min at 4 °C, and supernatant absorbance at 620 nm was read using a spectrophotometer. The concentration of dye in the extract was measured from a standard curve of Evans blue in formamide and normalized to retinal dry weight.

### 2.6 Mouse Retina Flow Cytometry

P17 mice were sacrificed, and their eyes were enucleated. The retina was dissected from surrounding ocular tissue and preserved in a cold Hanks balanced salt solution (HBSS). Four retina samples were placed into a microcentrifuge tube containing 1 mL of digestion solution (30U mL^−1^ Papain (Worthington, #LS003126), 5.5 mm L‐cysteine (Solarbio, #C0012), 1.1 mm EDTA (Invitrogen, #AM9260G) and 3 mg mL^−1^ DNaseI (Roche, #10104159001) in HBSS) and were incubated at 37 °C for 5 min. Gentle and repeated pipetting was performed to ensure thorough tissue digestion. Digested retinas after adding 10 mL of PBS+10% FBS. The suspension was filtered through a 40 µm cell strainer and centrifuged at 500 g for 5 min at 4 °C. The cell pellet was resuspended in 1 ml of PBS+2% FBS (staining buffer) in a 5 mL FACS tube. Cell counting was performed before staining. Stain for viability (live/dead stain) by diluting the fixable viability dye (FVS) (eBioscience, #65‐0865‐14) at 1:1000 in PBS and resuspend 1.0 × 10^6^ cells in diluted 100 µL of FVS solution. Incubate the samples for 7 min at 37 °C in the dark. Following staining, cells were washed, resuspended, and subjected to double CD31/VEGF staining. Cells were stained with 0.4 ug per million FITC‐conjugated anti‐mouse CD31 antibody in 100 µL volume, incubating for 30 min at 4 °C. Intracellular VEGF was stained by 10 µL PE‐conjugated VEGF antibody (R&D Systems, #23410) after fixation and permeabilization using a Fixation/Permeabilization kit (BD Biosciences, #554715). After washing, resuspend the cells in 300 µL stain buffer and cover with foil before analysis on the flow cytometer BD LSRFortessa (BD Biosciences). Data were processed with FlowJo (FlowJo.LCC)

### 2.7 Culture, Transfection, and Hypoxia Treatment of HUVECs

Human umbilical vein endothelial cells (HUVECs) were obtained from the American Type Culture Collection (ATCC, #CRL‐1730) and grown in DMEM containing 10% FCS and 1% antibiotic mixture (penicillin and streptomycin) at 37 °C under a humidified atmosphere of 5% CO_2_ and 20% O_2_. Cells were transfected for 24 h with a small interfering RNA (siRNA) targeting human AIP1 (GGGATAGGCTAAGGAGTAA; 40 nm), CASP8(CTGGACTACATTCCGCAAA;30nm), NLRP12(GCAGGAAATTCCGGCTCAT; 30 nm), or GSDMD (GCAGGAGCTTCCACTTCTA; 50 nm) as indicated to induce knockdown or with a pAd‐AIP1 plasmid (1 g) to induce AIP1 overexpression (all from Ribobio Co.) using Lipofectamine 3000 according to the manufacturer's instructions. A negative control (NC) siRNA or an empty vector (both from Ribobio) served as the controls. Transfected HUVECs were then cultivated in serum‐free DMEM within a portable three‐gas controlled incubator (Smartor 118) under 5% CO_2_ and 1% O_2_ (hypoxia) for 12 h at 37 °C. Cells cultured in a serum‐free medium under normoxia were used as controls. Other cells were treated with the CASP1 inhibitor YVAD (30 µm, Abcam) for 24 h before hypoxia treatment. Cells were then examined by immunostaining and tube formation assays.

### 2.8 Immunofluorescence Staining

The immunostaining protocol for whole‐mount retinas was described in a previous study by Tual‐Chalot et al.^[^ ^27^ ^]^ Eyes were removed and retinal whole‐mounts were prepared for staining with an antibody targeting isolectin B4‐Alexa 488 (1:100) to assess the retinal vasculature. 12 h after hypoxia exposure, cells treated as described were fixed with 4% paraformaldehyde at room temperature for 20 min, washed three times with 1× PBS, permeabilized with 0.3% Triton‐X‐100 for 5–15 min, blocked with 3% BSA for 1 h at room temperature, and incubated with primary antibodies targeting ASC, NLRP12, or CASP8 (1:100) as indicated at 4 °C overnight (all compounds in PBS). After at least three rinses with 1× PBS, cells were incubated in the dark with corresponding secondary antibodies (1:200) for 1 h at room temperature. Images were acquired using an Olympus fluorescence microscope.

### 2.9 HUVEC Tube Formation Assay

Angiogenesis μ‐Slides (ibidi, 81506) were coated with Matrigel (BD Biosciences, #356231) and placed in an incubator at 37 °C for 30 min to form a gel layer. After hypoxic treatment, HUVECs were suspended in VEC growth medium (PromoCell, #C‐22010) at 2 × 10^5^ cells mL^−1^, and then 50 µL was added to each well of the angiogenesis μ‐Slide. After incubation for 6 h at 37 °C, Matrigel‐induced tube formation was quantified under an Olympus fluorescence microscope.

### 2.10 Western Blotting

Proteins were extracted from HUVECs or retinal tissues, separated by SDS‐PAGE, and transferred onto polyvinylidene difluoride (PVDF) membranes. Membranes were blocked with 5% skimmed milk dissolved in TBST, incubated with the indicated primary antibodies at 4 °C overnight, and then incubated with appropriate secondary antibodies at 1:10000. Immunolabeled protein bands were visualized using an enhanced chemiluminescence kit (ECL, eBioscience) and captured using an Image Lab system (Bio‐Rad, USA).

### 2.11 Co‐Immunoprecipitation (co‐IP)

Cells were incubated in Cell Lysis Buffer (Cell Signaling Technology, #9803) containing a protease inhibitor cocktail for 15 min on ice, and raw lysates were centrifuged at 14 000 g for 30 min at 4 °C to obtain supernatants. After protein quantification, the supernatants were incubated with Protein A/G‐agarose beads (Abmart, #A10001) for 10 min at 4 °C to remove nonspecific proteins re‐centrifuged to remove the beads and incubated with the indicated antibody or mouse IgG overnight at 4 °C. A 20 µL volume of Protein A/G‐agarose was added, and the mixture was incubated for 3 h at 4 °C. The beads were isolated by centrifugation, resuspended in 60–80 µL of 2× sample buffer, and boiled at 100 °C for 5 min to remove pulled‐down protein complexes. Protein complexes were then separated by SDS‐PAGE gels and detected by WB.

### 2.12 Enzyme‐Linked Immunosorbent Assay (ELISA)

Concentrations of VEGFA in HUVEC supernatants were measured using a human VEGFA ELISA kit (ABclonal Biotechnology, #RK00023) according to the manufacturer's instructions.

### 2.13 Quantitative Real‐Time PCR (RT‐qPCR)

Total RNA was isolated from HUVECs or retinal tissues using TRIzol reagent (Invitrogen) and reverse transcribed into cDNA using FastKing gDNA Dispelling RT SuperMix (TIANGEN, #KR118‐02) according to the manufacturer's instructions. RT‐qPCR was performed using LightCycler 480 SYBR Green I Master mix (Roche) and a Light Cycler 480 Real‐Time PCR system with software version LCS480 1.5.1.62 according to the manufacturer's instructions. The primers used are listed in **Table** **3**.

**Table 3:** Primers used for PCR.

Human Gene	Primers
β‐actin	forward: 5′‐CTACCTCATGAAGATCCTCACCGA‐3′ reverse: 5′‐TTCTCCTTAATGTCACGCACGATT‐3′
AIP1	forward: 5′‐ GCAGGATGGTGATGGTTTGGT‐3′ reverse: 5′‐GGCAGTTCGTGGAGAAGTGGT‐3′
NLRP12	forward: 5′‐GAGCCAGCAGATAGGACCATT‐3′ reverse: 5′‐ CGACCTTTACCTGACCAACAA‐3′
CASP8	forward: 5′‐TTCAAAGGTCGTGGTCAAAGC‐3′ reverse: 5′‐AAAGCAAACCTCGGGGATACT‐3′
VEGFB	forward: 5′‐ ACTTGCACAGAGTGGTTAGAAAC‐3′ reverse: 5′‐GCCATCATCAAACAGGACAGA‐3′
VEGFR2	forward: 5′‐CATAGACATAAATGACCGAGGC‐3′ reverse: 5′‐CAAAGGGTGGAGGTGACTGAG‐3′
VEGFA	forward: 5′‐AAGGAGGAGGGCAGAATCAT‐3′ reverse: 5′‐ ATCTGCATGGTGATGTTGGA‐3′
Mouse Gene	Primers
β‐actin	forward: 5′‐GGGAAATCGTGCGTGAC‐3′ reverse: 5′‐AGGCTGGAAAAGAGCCT‐3′
AIP1	forward: 5′‐CTGGCACTTGAATAGGGTCTC‐3′ reverse: 5′‐CAGGGATAGGCTAAGGAGTAAG‐3′
NLRP12	forward: 5′‐GAATAGGAGACGGTCAGGAGC‐3′ reverse: 5′‐ GCAGGGAGTTGAATAGAAGCC‐3′
CASP8	forward: 5′‐ GTCACCGTGGGATAGGATACA‐3′; reverse: 5′‐AGACATAACCCAACTCCGAAAA‐3′
VEGFB	forward: 5′‐TGGCAGCTCTGGGAGATAAAA‐3′ reverse: 5′‐ACAGGCGAACCTCCTCAGTCT‐3′
VEGFR2	forward: 5′‐TGTTCTTGTTCTCGGTGATGT‐3′ reverse: 5′‐ GCCTCCACTGTTTATGTCTATG‐3′
VEGF‐A	forward: 5′‐CAGGCTGCTGTAACGATGAA‐3′ reverse: 5′‐GCATTCACATCTGCTGTGCT‐3′

### 2.14 Statistics

All data are presented as the mean ± SD of at least three independent experiments. Data were pre‐processed by normalizing to control conditions. Differences among three or more group means were analyzed using one‐way ANOVA followed by Dunnett's post hoc tests. The independent‐sample *t*‐test was used to assess the difference between the two group means. All statistical calculations were performed using *p* < 0.05 (two‐tailed) was considered statistically significant for all tests. Statistical analyses were performed using GraphPad Prism 8.

### 2.15 Availability of Data and Material

The data supporting these findings are available from the corresponding author upon reasonable request. The scRNA‐seq data were deposited in the National Center for Biotechnology Information Gene Expression Omnibus (GEO) (GSE173079).

## 3 Results

### 3.1 AIP1 Expression was Downregulated in Retinal VECs of OIR as Revealed by scRNA‐seq

Pathologic angiogenesis induced by hypoxia is a hallmark of ischemic retinopathy including DR and ROP, characterized by retinal VECs dysfunction, vascular regression, and compensatory neovascularization.^[^ ^10^, ^28^ ^]^ Retinal inflammation plays a significant role in vascular pathology, with VECs actively promoting inflammation via NF‐κB‐mediated transcription of pro‐inflammatory cytokines and facilitating vascular leakage, creating a vicious cycle that further exacerbates endothelial dysfunction.^[^ ^7^, ^8^, ^29^ ^]^

**Figure 1:**
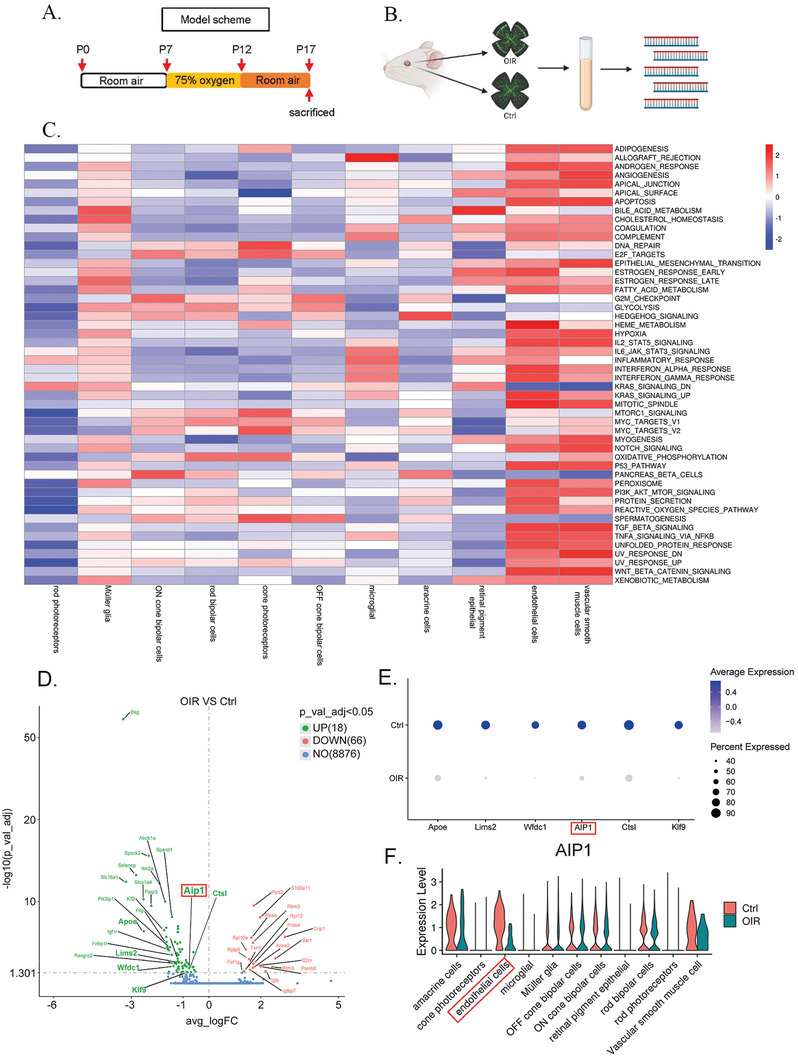
scRNA‐seq of retinas from OIR mice highlights the angiogenesis and inflammation in vascular endothelial cells with AIP1 as a significant differential molecule. (A) Experimental design for establishing the OIR model. Mouse pups were randomly assigned to either a control group or an OIR group exposed to 75% oxygen from P7 to P12, followed by 5 days of normoxia. (B) The scRNA‐seq procedure for gene expression profiling of retinal cells from control and OIR model mice (created with Biorender.com). (C) GSVA analysis characterized gene expression across various clusters in the retina of OIR mice. Notable gene sets, including inflammatory response, were implicated in retinal VECs. Columns represent different cell subpopulations, while rows indicate specific gene sets. The colors red and blue represent gene activation and suppression respectively. (D) The volcano plot demonstrated significant downregulation of AIP1 in the VECs in the retina of OIR mice. Each red dot represents a gene with an adjusted *p* < 0.05 (Wald test) and log2 (fold change) ≥ 0.25 (upregulated in OIR). Each green dot represents a gene with adjusted *p* < 0.05 and log2 (fold change) ≤ −0.25 (downregulated in OIR). (E) The dot plot showed differentially expressed genes involved in the negative regulation of the angiogenesis pathway between the OIR and control groups, with AIP1 prominently ranked among them. (F) The violin plot illustrated the expression levels of AIP1 across multiple retinal cell types in OIR mice compared to controls. Notably, endothelial cells exhibited the most pronounced downregulation of AIP1 in the OIR group.

To generate a detailed molecular atlas of retinal VECs in pathologic retinal angiogenesis, we compared droplet‐based scRNA‐seq (10×Genomics) data between retinas from OIR mice, a model mimicking ischemic retinopathy, and untreated control mice. Expression profiles of 38 542 dissociated retinal cells were collected from three independent samples per group with good‐quality reads for all samples (**Figure** **1**A,B). Marker gene expression in each cluster was represented as violin plots (Figure S1A, Supporting Information). Differential expression analysis was performed for each cluster, and subpopulations were defined according to published articles (**Table** **4**). The VEC cluster was defined by the higher expression of *Cldn5* compared to all other cell types.

**Table 4:** Marker genes for cell types.

Cell type	Marker genes
Rod photoreceptors	*Rho*, *Nrl*, *Nr2e3* ^[^ ^53^ ^]^
Müller glia	*Vim*, *Slc3a2*, *Glul*, *Rlbp1* ^[^ ^53^ ^]^
Rod bipolar cell	*Vsx2* ^+^, *Prkca* ^+[^ ^54^ ^]^
Cone photoreceptors	*Opn1mw*, *Arr3* ^[^ ^53^ ^]^
ON cone bipolar	*Isl1* ^+^, *Grm6* ^+^, *Prkca* ^−[^ ^54^ ^]^
OFF cone bipolar	*Grik1* ^+^, *Prkca* ^−[^ ^54^ ^]^
Microglial	*Cx3cr1* ^[^ ^55^ ^]^
Amacrine cells	*Tfap2a, Pax6, Gad1* ^[^ ^53^ ^]^
Retinal pigment epithelial	*Rpe65, Rdh5, Rdh10, Dct* ^[^ ^56^ ^]^
Vascular Endothelial cells	*Cldn5* ^[^ ^53^, ^55^ ^]^
Vascular smooth muscle cell	*Myh11, Tpm2, Myl9, Acta2, Tagln* ^[^ ^57^ ^]^

Subsequently, we conducted a Gene set variation analysis (GSVA) analysis of Hallmark gene sets for each cell type. We found that in OIR, the activity of pathways related to hypoxia and angiogenesis was notably increased in VECs and vascular smooth muscle cells (Figure 1C). RNV originates from VECs dysfunction,^[^ ^9^ ^]^ making VEC dysfunction an early indicator for predicting and identifying retinal neovascularization.^[^ ^10^ ^]^ It is noteworthy that multiple inflammatory pathways have been identified as significantly enriched within VECs. This observation suggests the potential existence of key molecular mediators that regulate angiogenesis and inflammation concurrently (Figure 1C). We further analyzed retinal VECs populations and observed differences between the OIR group and the control group. Compared to the control group, 18 genes were upregulated and 66 genes were downregulated in the OIR group. We further analyzed the expression‐based phenotypic heterogeneity of VECs between OIR and control groups. Among these downregulated differentially expressed genes, the most distinctive ones (*Poe*, *Lims2*, *Wfdc1*, *AIP1*, *Ctsl*, and *Klf9*) were found to be associated with the regulation of angiogenesis (Figure 1D,E). According to the Gene Ontology (GO) database, the upregulated and downregulated genes and associated GO pathways were shown in the supplementary figure (Figure S1B,C, Supporting Information).

AIP1 is a molecule involved in angiogenesis, and previous studies have reported that AIP1 directly inhibits the VEGF signaling pathway and mediates TLR4‐related inflammatory pathways.^[^ ^30^ ^]^ In our study, we found that AIP1 signaling was downregulated in retinal VECs of the OIR group. Interestingly, no significant changes in AIP1 signaling were observed in other cell types (Figure 1F). This suggests a specific reduction of AIP1 signaling in retinal VECs of OIR mice. Therefore, we hypothesize that in the retinas of OIR model mice, AIP1 may function in VECs and be associated with disease pathogenesis, potentially involving inflammatory processes.

### 3.2 AIP1 is an Endogenous Inhibitor of VEGFA‐Associated Signaling that Restricts Disease Progression in OIR

**Figure 2:**
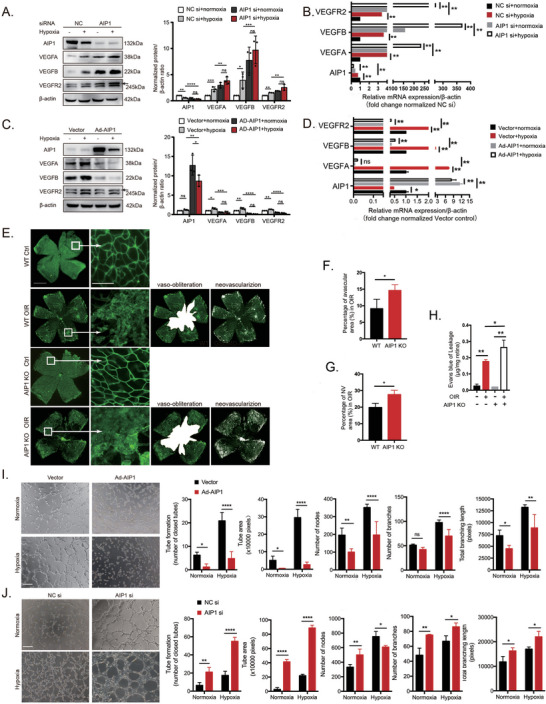
The anti‐angiogenic factor AIP1 protects against VECs dysfunction induced by the OIR and hypoxia in vitro. (A and B) Protein and mRNA levels of VEGF‐associated signaling components in HUVECs transfected with control siRNA or AIP1 siRNA (n = 3 cultures/group). (C and D) Protein and mRNA levels of AIP1, VEGFA, VEGFB, and VEGFR2 in control HUVECs and HUVECs transfected with AIP1 overexpression plasmid (n = 3 cultures/group). (E) Representative images of the retinal flat mounts stained with IB4 from WT and AIP1‐deficient mice (n = 6 mice/group). Vaso‐obliteration and neovascularization are indicated by the white area and white dots, respectively. Scale bar: 1 mm for 4× images and 100 µm for 40× images. Avascular area (F), neovascular area (G), and fluid permeability measured by Evans blue dye (H) were quantified. (I) Representative images and quantitative measurements of tube formation assays using HUVECs transfected with empty vector or AIP1 overexpression plasmid before hypoxia exposure. (J) Representative images and quantitative measurements from in vitro tube formation assays using HUVECs transfected with control siRNA or AIP1 siRNA before hypoxia exposure. Scale bar: 200 µm. NV: neovascularization. Data are presented as the mean ± SD. ^*^ *p* < 0.05, ^**^ *p* < 0.01, ^***^ *p* < 0.001, ^****^ *p* < 0.0001, ns, not significant. Statistical analyses were performed using GraphPad Prism software, with one‐way ANOVA followed by Dunnett's post hoc tests and independent‐sample t‐test.

Consistent with the scRNA‐seq results showing reduced expression of AIP1 in retinal VECs, we also observed a significant reduction in AIP1 expression among HUVECs exposed to hypoxia in vitro compared to control HUVECs at both protein and mRNA levels. We further examined the association between AIP1 and VEGFA signaling in HUVECs. VEGFA, VEGFB, and VEGFR2 protein and mRNA expression levels were suppressed by the overexpression of exogenous AIP1. Conversely, AIP1 knockdown increased the protein and mRNA levels of VEGFA, VEGFB, and VEGFR2 in HUVECs (**Figure** **2**A–D). Moreover, AIP1‐deficient mice exhibited retinal vascular abnormalities compared to WT mice, as revealed by direct vessel visualization through IB4 immunofluorescence staining of whole‐mount preparations. Strikingly, both vaso‐obliteration and neovascularization were dramatically increased by the genetic deletion of AIP1 (Figure 2E–G). In addition, AIP1‐knockout mice exhibited more severe vascular leakage than WT mice as measured by the Evans blue assay (Figure 2H). Collectively, these findings suggest that AIP1 may help maintain appropriate VECs functions under hypoxia, thereby mitigating vascular pathology. To test these protective effects, we conducted tube formation assays using HUVECs with AIP1 knockdown or overexpression. Transfection with an overexpression vector (pAd‐AIP1 plasmid) significantly inhibited hypoxia‐induced tube formation, whereas siRNA‐mediated AIP1 knockdown markedly enhanced tube formation (Figure 2I,J). These results suggest that reduced endogenous AIP1 in the OIR retina may contribute to pathologic neovascularization and disease progression. This suggests that AIP1 may protect against OIR by negatively regulating angiogenesis‐associated VEGF signaling.

### 3.3 Hypoxia‐Induced AIP1 Reduction Enhances the Activation of NLRP12 and Caspase‐8 Inflammasomes

**Figure 3:**
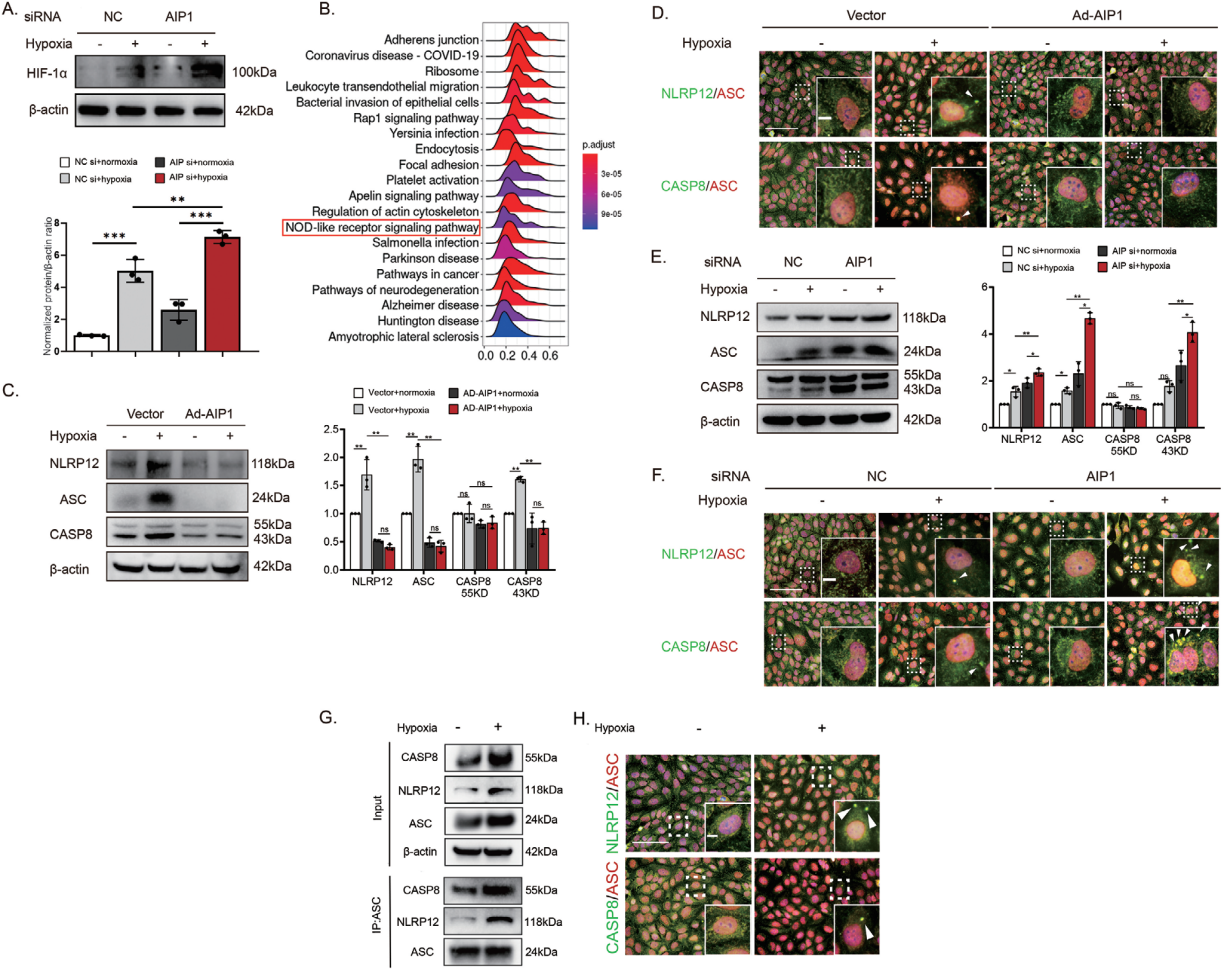
Identification of the NLRP12‐ASC‐CASP8 inflammasome and its suppression by AIP1. (A) Elevated HIF‐1α protein levels in HUVECs transfected with AIP1 siRNA under hypoxia conditions (n = 3 cultures/group). (B) The ridge plot represents a single‐gene GSEA analysis based on AIP1 in VECs cluster, showing a marked association between the NOD‐like receptor signaling pathway and AIP1 in VECs. (C) Decreased NLRP12‐ASC‐CASP8 inflammasome protein levels in HUVECs overexpressing AIP1 (n = 3 cultures/group). (D) Representative images showing the assembly and activation of the NLRP12‐ASC‐CASP8 inflammasome in HUVECs overexpressing AIP1. Arrows indicate the inflammasome complex. Scale bar: 200 µm. (E and F) Activation of the NLRP12–CASP8 inflammasome as measured by dual immunofluorescence staining and immunoblotting in HUVECs transfected with AIP1 siRNA. Arrows indicate the inflammasome complex. Scale bar: 200 µm. (G) The NLRP12‐ASC‐CASP8 inflammasome was identified by co‐IP assay with an anti‐ASC antibody, and immunoblotting was used to detect CASP8, NLRP12, and ASC. (H) Representative images of immunofluorescence staining for NLRP12, ASC, and CASP8 in HUVECs exposed to hypoxia. Arrows indicate the inflammasome assembly. Scale bar: 200 µm. Data are presented as the mean ± SD. ^*^ *p* < 0.05, ^**^ *p* < 0.01, ^***^ *p* < 0.001, ^****^ *p* < 0.0001, ns, not significant. Statistical analyses were performed using GraphPad Prism software, with one‐way ANOVA followed by Dunnett's post hoc tests.

AIP1 has been shown to inhibit TLR4 in LPS‐stimulated VECs, indicating a potential regulatory role in inflammation.^[^ ^31^, ^32^ ^]^ Additionally, our previous study showed that TLR4 promotes activation of the NLRP12 inflammasome and caspase‐8.^[^ ^5^ ^]^ We also found that NLRP12 collaborates with NLRC4 to mediate pyroptosis in microglia, a process dependent on the hypoxia‐inducible factor‐1α (HIF‐1α) signaling pathway.^[^ ^33^ ^]^ Knockdown of AIP1 resulted in an elevation of HIF‐1α protein expression levels (**Figure** **3**A). Furthermore, we conducted a single GSEA based on AIP1 in the VECs cluster from scRNA‐seq. The ridge plot revealed that the NOD‐like receptor signaling pathway was among the top 20 enriched biological processes in genes correlated with AIP1 (Figure 3B). In this study, we further delineated the precise effects of AIP1 on inflammatory angiogenesis and its underlying mechanisms by genetic ablation or overexpression of AIP1 in HUVECs. Immunoblotting revealed that the overexpression of AIP1 significantly reduced the expression levels of NLRP12, ASC, and cleaved CASP8 (Figure 3C). Immunofluorescence staining further showed that AIP1 overexpression disrupted the co‐localization of NLRP12–ASC and CASP8–ASC observed in control cells, strongly suggesting that AIP1 suppresses the formation/activation of NLRP12 and caspase‐8 inflammasomes and downstream inflammatory signaling pathways (Figure 3D). Conversely, AIP1 knockdown enhanced NLRP12, ASC, and CASP8 expression and co‐localization (Figure 3E,F). We confirmed the assembly of NLRP12 and CASP8 into inflammasomes through co‐IP assays and dual immunofluorescence staining. In the co‐IP assays, the co‐precipitation of CASP8 and NLRP12 with ASC was markedly enhanced under hypoxia (Figure 3G). Additionally, a significant increase in the co‐localization of NLRP12 and ASC, as well as CASP8 and ASC, was detected through immunofluorescence staining (Figure 3H). These results suggest that AIP1 not only participates in VEGF signaling but also regulates inflammatory vasculopathy and angiogenesis by suppressing the assembly of the novel NLRP12‐ASC‐caspase‐8 complex.

### 3.4 The NLRP12–CASP8‐ASC Inflammasome Promotes Vaso‐Obliteration, Neovascularization, and Tube Formation

**Figure 4:**
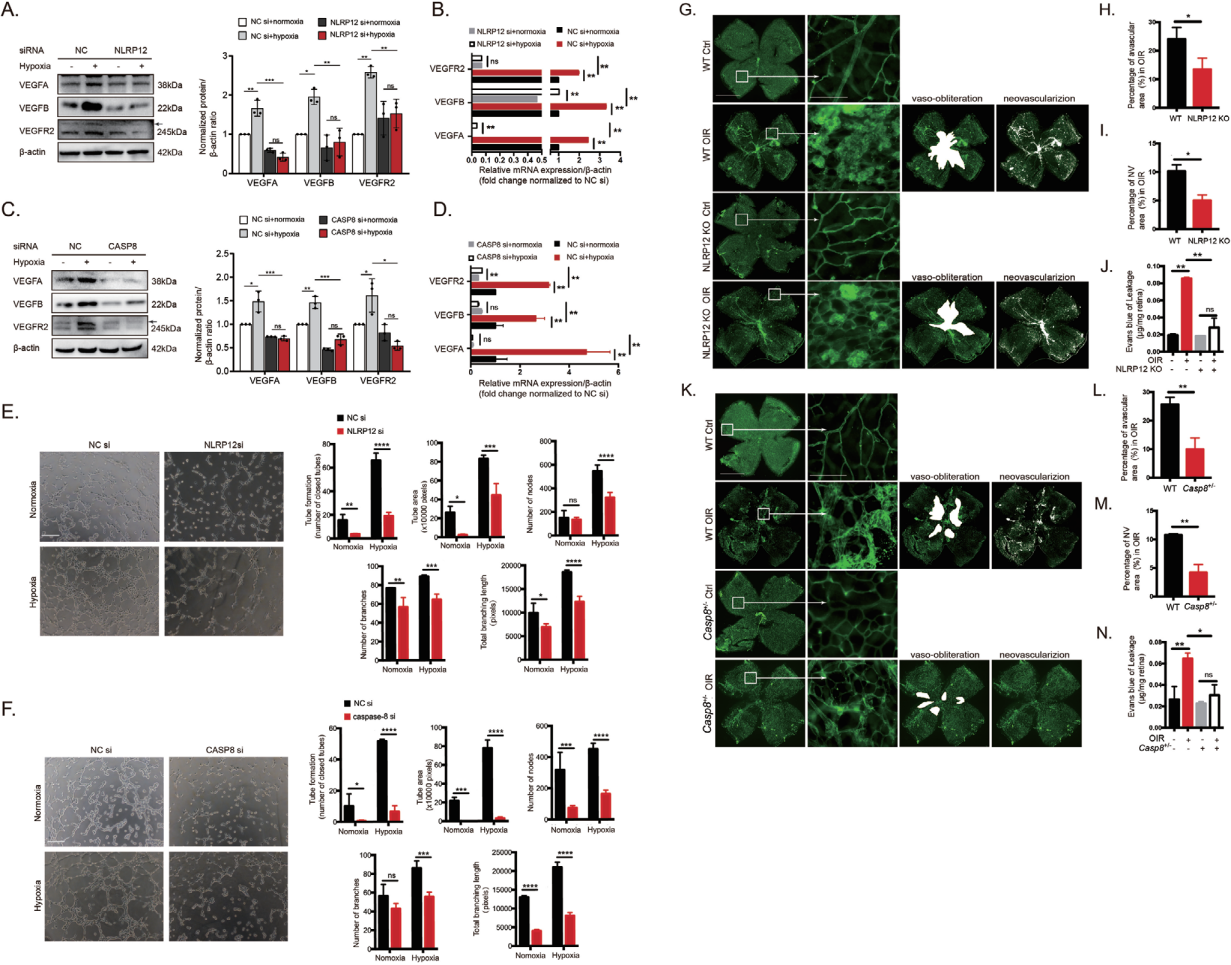
The NLRP12–CASP8 inflammasome enhances VEGF signaling. (A and B) NLRP12 knockdown reduced the protein and mRNA levels of VEGFA, VEGFB, and VEGFR2 in HUVECs exposed to hypoxia (n = 3 cultures/group). (C and D) CASP8 knockdown reduced the protein and mRNA levels of VEGFA, VEGFB, and VEGFR2 in HUVECs exposed to hypoxia (n = 3 cultures/group). (E and F) Representative images and quantitative measurements of tube formation assays using HUVECs transfected with NC siRNA, NLRP12 siRNA, or CASP8 siRNA before hypoxia exposure. Scale bar: 200 µm. (G to N) Representative images and quantitative measurements of the retinal flat mounts stained with IB4 from WT, NLRP12 KO (G), and CASP8 ^+/−^ (K) mice (n = 6 mice/group). Vaso‐obliteration and neovascularization are indicated by the white area and white dots, respectively. Scale bar: 1 mm for 4× images and 100 µm for 40× images. Analyses of whole‐mount retinal immunofluorescence images, including nonperfused areas (H,L), new vessel tuft formation (I,M), and Evan blue dye permeability (J,N). Data are presented as the mean ± SD. ^*^ *p* < 0.05, ^**^ *p* < 0.01, ^***^ *p* < 0.001, ^****^ *p* < 0.0001, ns, not significant. Statistical analyses were performed using GraphPad Prism software, with one‐way ANOVA followed by Dunnett's post hoc tests and the independent‐sample t‐test.

Aberrant VEGF signaling is central to the pathologic angiogenesis observed during tumor progression and in the development of retinal vascular diseases,^[^ ^1^ ^]^ Thus, we examined the potential effects of this novel noncanonical NLRP12‐CASP8‐ASC inflammasome on VEGF signaling. The elimination of NLRP12 or CASP8 in HUVECs markedly diminished both protein and mRNA levels of VEGFA, VEGFB, and VEGFR2 (**Figure** **4**A–D), suggesting that this inflammasome promotes VEGF signaling and angiogenesis. We then conducted endothelial tube formation assays in vitro using transfected HUVECs. The number and length of hypoxia‐induced tube‐like structures were markedly reduced in cultures transfected with NLRP12 siRNA (Figure 4E) or CASP8 siRNA (Figure 4F), suggesting a previously unknown role of NLRP12 and CASP8 in retinal vasculopathy and neovascularization. To further delineate the effects of NLRP12 and CASP8 on angiogenesis, IB4 immunofluorescence staining revealed that nonperfused areas and neovascular tuft areas were significantly smaller in NLRP12^−/−^ mice (Figure 4G–I) and CASP8^+/−^ mice (Notably, heterozygotes were used because total ablation is embryonic lethal) (Figure 4K–M) following OIR. Likewise, genetic ablation of NLRP12 or CASP8 suppression also mitigated the increase in retinal vascular permeability following OIR compared to WT littermates, as measured by Evans blue assay (Figure 4J–N). Taken together, these data provide persuasive evidence that activation of the newly identified NLRP12‐CASP8 inflammasome exacerbates hypoxia‐induced vaso‐obliteration and neovascularization during OIR by driving VEGF signaling.

### 3.5 The NLRP12–CASP8 Inflammasome Promotes Inflammatory Cytokine Generation and GSDMD‐Mediated Pyroptosis of VECs During OIR

**Figure 5:**
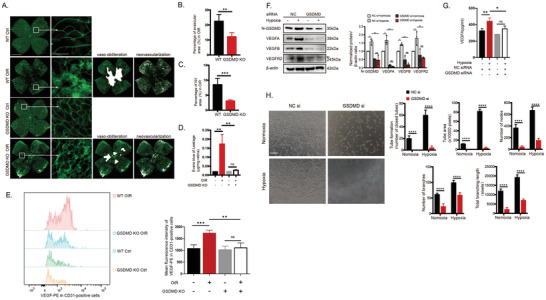
GSDMD‐mediated pyroptosis and VEGF signaling of VECs drive OIR. (A) Retinal vasculature in WT and GSDMD KO mice was visualized using IB4 immunofluorescence staining (n = 6 mice/group). Scale bar: 1 mm for 4× images and 100 µm for 40× images. (B–D) Analysis of whole‐mount retinal immunofluorescence images, including avascular areas (B), neovascular tuft areas (C), and Evans blue dye permeability (D). (E) Flow cytometry analysis of VEGF‐PE expression in CD31‐positive cells (vascular endothelial cell marker) in the retina of WT and GSDMD KO mice (n = 6 mice/group). (F) GSDMD knockdown reduced VEGFA, VEGFB, and VEGFR2 protein levels in HUVECs exposed to hypoxia (n = 3 cultures/group). (G) VEGFA levels in the supernatant of HUVECs exposed to hypoxia were measured using ELISA (n = 3 cultures/group). (H) Representative images and quantitative measurements of tube formation assays using HUVECs transfected with NC siRNA and GSDMD siRNA before hypoxia exposure. Scale bar: 200 µm. Data are presented as the mean ± SD. ^*^ *p* < 0.05, ^**^ *p* < 0.01, ^***^ *p* < 0.001, ^****^ *p* < 0.0001, ns, not significant. Statistical analyses were performed using GraphPad Prism software, with one‐way ANOVA followed by Dunnett's post hoc tests and the independent‐sample *t*‐test.

Our previous study showed that CASP8 and NLRP12 promote microglia pyroptosis, which mediates ischemic retinopathy.^[^ ^5^, ^34^ ^]^ However, it remains unclear whether pyroptosis is involved in the inflammatory angiogenesis. Therefore, we investigated the effects of pyroptosis on RNV by comparing whole‐mount immunofluorescence IB4 staining patterns between WT and GSDMD KO mice following OIR. Genetic deletion of GSDMD significantly reduced the avascular area and pathologic neovascular tufts compared to WT following OIR (**Figure** **5**A–C). Furthermore, GSDMD‐KO mice exhibited a lower Evans blue dye leakage rate following OIR injury compared to WT mice (Figure 5D), suggesting that GSDMD deletion and the subsequent inhibition of pyroptosis attenuates retinal VECs dysfunction and preserves VECs barrier integrity under OIR conditions.

Angiogenesis and the maintenance of vascular homeostasis require the dynamic modulation of VECs through various local and systemic signaling factors.^[^ ^35^, ^36^ ^]^ The VEGF signaling pathway is pivotal in promoting retinal neovascularization. Pyroptosis may represent a critical pathway leading to VECs dysfunction under various pathological conditions.^[^ ^37^, ^38^ ^]^ The potential link between GSDMD‐mediated pyroptotic pore formation and VEGF release warrants further investigation, suggesting an intriguing interplay between inflammatory cell death mechanisms and angiogenic responses. Therefore, we further explored the alterations in VEGF expression within VECs following GSDMD gene depletion in the GSDMD KO mice model of OIR. Retinas were processed into single‐cell suspensions and dual staining of CD31 (a marker of VECs) and VEGF was used to assess VEGF expression in CD31‐positive cells. Flow cytometry revealed a decrease in VEGF levels in GSDMD KO OIR mice compared to the WT OIR mice (Figure 5E). Selective knockdown of GSDMD in HUVECs significantly decreased the expression of VEGF signaling components VEGFA, VEGFB, and VEGFR2 (Figure 5F) and reduced VEGFA secretion (Figure 5G). This suggests that GSDMD normally promotes VEGF signaling and the extracellular secretion of VEGFA from VECs, thereby driving RNV. Matrigel assays demonstrated that GSDMD knockdown in HUVECs significantly reduced hypoxia‐induced tube formation and network complexity (Figure 5H). This suggests that GSDMD‐mediated pyroptosis of VECs contributes to vaso‐obliteration and neovascularization.

**Figure 6:**
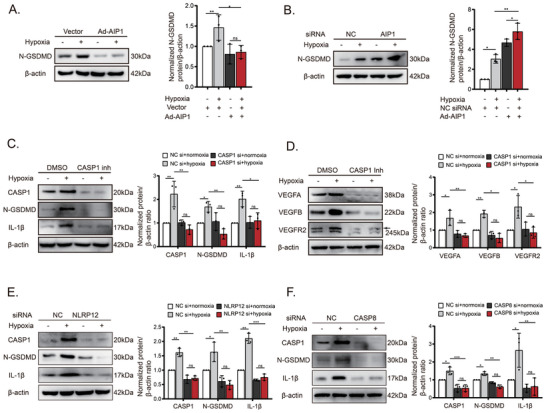
The NLRP12–CASP8 inflammasome promotes GSDMD‐mediated pyroptosis and VEGF expression of VECs. (A and B) Cleavage of GSDMD was detected by Western blot in HUVECs with AIP1 overexpression or AIP1 knockdown, respectively (n = 3 cultures/group). (C and D) Components of the pyroptotic‐ and VEGFA‐associated pathways were assayed by Western blot following CASP1 inhibition (n = 3 cultures/group). (E and F) Western blot analysis of pyroptotic hallmarks, including CASP1, GSDMD cleavage and IL‐1β maturation, after NLRP12 or CASP8 knockdown (n = 3 cultures/group). Data are presented as the mean ± SD. ^*^ *p* < 0.05, ^**^ *p* < 0.01, ^***^ *p* < 0.001, ns, not significant. Statistical analyses were performed using GraphPad Prism software, with one‐way ANOVA followed by Dunnett's post hoc tests.

**Figure 7:**
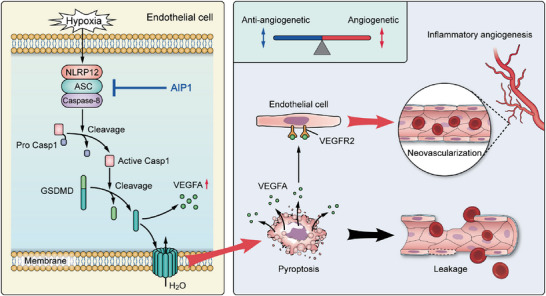
A new model of ROP pathogenesis involving AIP1 downregulation, NLRP12–CASP8inflammasome activation, GSDMD‐dependent VECs pyroptosis, excessive VEGFA production, and neovascularization. All images were generated by the authors.

Finally, we investigated the mechanisms underlying pyroptosis and its role in inflammatory angiogenesis. AIP1 overexpression markedly suppressed hypoxia‐induced GSDMD cleavage, while AIP1 downregulation increased N‐terminal GSDMD expression (**Figure** **6**A,B). CASP1 inhibition significantly reduced GSDMD cleavage and the expression of VEGF signaling components in HUVECs, suggesting that CASP1‐induced, GSDMD‐dependent pyroptosis drives VEGF signaling and promotes neovascularization in OIR (Figure 6C,D). Knockdown of either NLRP12 or CASP8 reduced CASP1, GSDMD, and cleaved IL‐1β expression in HUVECs (Figure 6E,F), indicating that hypoxia‐induced activation of the noncanonical NLRP12‐ASC‐CASP8 inflammasome triggers CASP1‐driven, GSDMD‐dependent pyroptosis and IL‐1β maturation. In summary, these findings uncover a novel mechanism for inflammatory angiogenesis. Reduced levels of the angiogenesis inhibitor AIP1 trigger the activation of the NLRP12‐CASP8 inflammasome through a caspase‐1‐dependent pathway. This, in turn, leads to VECs pyroptosis and enhances VEGF signaling, ultimately promoting neovascularization (**Figure** **7**).

## 4 Discussion

Pathogenic retinal angiogenesis is central to the pathophysiology of many vision‐threatening diseases, characterized by new blood vessel formation surrounding avascular areas.^[^ ^39^ ^]^ This pathologic neovascularization is thought to be associated with dysfunctional VEGF signaling. Thus, binding VEGFA to decoy receptors or using VEGF‐neutralizing antibodies are common therapeutic approaches in clinical practice.^[^ ^40^, ^41^, ^42^ ^]^ In addition to paracrine VEGF signaling, autocrine VEGFA is pivotal for the maintenance of VECs survival,^[^ ^43^ ^]^ though the mechanisms underlying endothelial autocrine VEGFA release remain elusive. Here, using a murine OIR model with retinal vaso‐obliteration and neovascularization induced by oxygen‐dependent retinal vascular injury and occlusion, we revealed novel mechanisms responsible for autocrine VEGFA release and endothelial dysfunction, identifying previously unknown therapeutic targets for intervention.

Although anti‐VEGF strategies are a promising treatment for ocular neovascularization, they have critical limitations in improving vision loss and regressing blood vessels due to VEGF‐induced pathological vessel maturation. Accumulating evidence indicates that angiogenesis is often associated with inflammation.^[^ ^30^, ^44^, ^45^ ^]^ However, the precise mechanisms of inflammatory angiogenesis are still obscure. Herein, we characterized the differences in retinal cell gene expression profiles between OIR and untreated mouse pups using scRNA‐seq, showing that neovascularization results from an imbalance between pro‐angiogenic and anti‐angiogenic factors, based on functional annotation analysis. We found that AIP1 was specifically expressed in retinal VECs and substantially downregulated in OIR retinas compared to untreated controls. Furthermore, AIP1 acts as a critical endogenous inhibitor of VEGF‐induced neovascularization by directly binding to VEGFR2 complex.^[^ ^14^, ^30^ ^]^ Consistent with this function, AIP1‐knockout mice exhibited more severe nonperfusion injury and angiogenesis in a murine OIR model, suggesting that the AIP1 downregulation during OIR may induce an imbalance between pro‐and anti‐angiogenic processes, leading to excessive retinal neovascularization. Conversely, AIP1 overexpression significantly inhibited VEGF‐related signaling in OIR, consistent with previous studies.^[^ ^14^, ^30^, ^46^ ^]^ A reduction in AIP‐1 in LPS‐stimulated ECs has been shown to augment pro‐inflammatory TLR4‐MyD88 signaling, highlighting AIP1 as a potential molecular link between inflammation and angiogenesis.^[^ ^47^ ^]^ Here, we provide evidence for this function by demonstrating that AIP1 suppresses the NLRP12 and CASP8 inflammasomes and GSDMD‐dependent pyroptosis. Further investigation is warranted to delineate the specific inflammasome components that interact with AIP1 and the mechanisms involved. Proximity labeling techniques, which excel at capturing transient and weak interactions, particularly when using AIP1 as a bait protein, offer a powerful strategy to map the local interactome and provide deeper insights into these dynamic molecular associations. In a previously published study on corneal alkali burn, we found that AIP1 regulated neovascularization primarily by influencing NOX4‐driven ROS production and the imbalance between NLRP3 and NLRP6 inflammasomes.^[^ ^48^ ^]^ This suggests that the mechanisms by which AIP1 regulates inflammasomes and pyroptosis differ depending on the cell type and the nature of the pathological damage involved. The proximity labeling technique is a powerful tool for studying the AIP1 interactome under various pathological conditions, helping to uncover cell‐type‐specific and pathology‐specific AIP1‐mediated protein networks. Moreover, GSDMD‐dependent pyroptosis of VECs resulted in enhanced VEGFA release, suggesting that AIP1 might act as a dual‐function suppressor of angiogenesis by reducing both VEGFR activity and VEGF ligand release. Recently, increasing attention has been given to the role of GSDMD in mediating barrier disruption, particularly in endothelial cells. Studies have highlighted how GSDMD activation compromises endothelial barrier integrity, leading to increased vascular permeability and tissue damage.^[^ ^49^ ^]^

This study provides the evidence that the pyroptosis effector GSDMD contributes to hypoxia‐induced blood vessel obstruction, pathogenic angiogenesis, and increased vascular permeability during OIR development. GSDMD knockdown in HUVECs reduced both VEGFA production and secretion, supporting the idea that VEC pyroptosis is a major contributor to VEGFA hyperactivity and angiogenesis in OIR. This finding suggests a novel mechanism mediating inflammatory angiogenesis, namely GSDMD‐dependent VECs pyroptosis under hypoxia resulting in unregulated VEGFA release and the autocrine activation of surviving VECs, with the ensuing enhancement of VECs viability, migration, and angiogenesis. Unlike the pro‐angiogenic VEGFR2, VEGFR1 regulates cell survival rather than angiogenesis and has a stronger affinity for VEGFA than VEGFR2.^[^ ^50^ ^]^ VEGFB is believed to indirectly promote angiogenesis by occupying VEGFR1‐binding sites, thus allowing VEGFA to bind VEGFR.^[^ ^11^ ^]^ In our study, VEGFB and VEGFR2 expression levels were markedly reduced by GSDMD knockdown, suggesting that GSDMD mediates both indirect and direct pro‐angiogenic signaling.

The NLRP3 inflammasome has been implicated in the neovascularization associated with wet AMD and DR,^[^ ^22^, ^51^ ^]^ but it remains unclear whether the newly identified NLRP12 sensor assembles and functions as an inflammasome in angiogenesis. WB, co‐IP and immunolocalization experiments revealed that NLRP12 and CASP8 are linked by the adaptor protein ASC to form an atypical inflammasome complex activated by hypoxia. Activation of this NLRP12‐CASP8 inflammasome promoted the CASP1‐dependent cleavage of GSDMD, inducing VECs pyroptosis. Furthermore, genetic ablation of NLRP12 or suppression of CASP8 expression reduced the formation of nonperfused and neovascular areas, vascular permeability, and VEGF signaling factor expression in the OIR model, suggesting that this atypical NLRP12‐CASP8 inflammasome could be an effective therapeutic target for RNV. Tisch et al. also reported that the loss of CASP8 in VECs reduces pathological neovascularization in ROP.^[^ ^52^ ^]^ We therefore infer that hypoxia‐induced NLRP12‐CASP8 inflammasome activation triggers GSDMD cleavage to drive vascular endothelial pyroptosis. The membrane pores formed by cleaved N‐terminal GSDMD fragments allow excessive VEGFA secretion, disrupting endothelial barrier integrity and increasing fluid leakage. Additionally, the spread of VEGFA subsequently amplifies neovascularization.

Our study showed that the ablation of CASP8/AIP1 significantly delayed normal vascularization in the retina. Our findings also provided evidence that the pyroptosis effector GSDMD, and its upstream initiator NLRP12, modulated normal retinal vascular development (Figure S2, Supporting Information). However, the underlying mechanisms require further investigation.

In summary, this study reveals a novel mechanism for pathogenic neovascularization in RNV. Briefly, NLRP12‐CASP8‐induced, GSDMD‐dependent VECs pyroptosis results in VEGFA release and promotes RNV. Thus, the NLRP12‐CASP8‐GSDMD axis provides a molecular link between inflammation and angiogenesis. Conversely, AIP1 protects against retinopathy by negatively regulating the NLRP12‐CASP8 inflammasome and pyroptosis, thereby suppressing inflammatory cascades and VEGF signaling. Thus, AIP1 is a promising protective factor against RNV. Comprehensive future studies are needed to determine how to effectively reduce pathologic neovascularization without affecting normal vascular survival and homeostasis.

## Conflict of Interest

The authors declare no conflict of interest.

## Author Contributions

Y.L., Y.S., D.X., and H.C. are co‐authors. Y.L. and W.C. performed conceptualization, data curation, formal analysis, acquired funding acquisition, investigation, methodology, project administration, resources, software, supervision, validation, visualization, writing‐original draft, writing‐review, and editing. Y.S. performed software, investigation, methodology, writing‐review, and editing. D.X. performed data curation, formal analysis, methodology, validation, visualization, writing‐review, and editing. H.C. performed conceptualization, formal analysis, investigation, methodology, writing‐original draft. Q.Z. performed data curation, formal analysis. S.Z. and F.W. performed supervision, writing‐review, and editing. J.O. performed acquired funding acquisition, supervision, writing‐review, and editing. M.Z. and L.S. performed data curation, formal analysis, methodology, validation. X.L. and W.W. performed supervision, writing‐review, and editing.

## Supporting information



Supporting Information

## Data Availability

The data that support the findings of this study are available from the corresponding author upon reasonable request.
