# Supplementary material for: AIP1 Regulates Ocular Angiogenesis Via NLRP12‐ASC‐Caspase‐8 Inflammasome‐Mediated Endothelial Pyroptosis
Source: Adv Sci (Weinh). 2024 Nov 11;11(47):2405834. doi: 10.1002/advs.202405834 (PMC11653709; doi:10.1002/advs.202405834)
Supplement: Supplementary file 1 — Supporting Information [file ADVS-11-2405834-s001.docx]

**Supplementary Material**

**
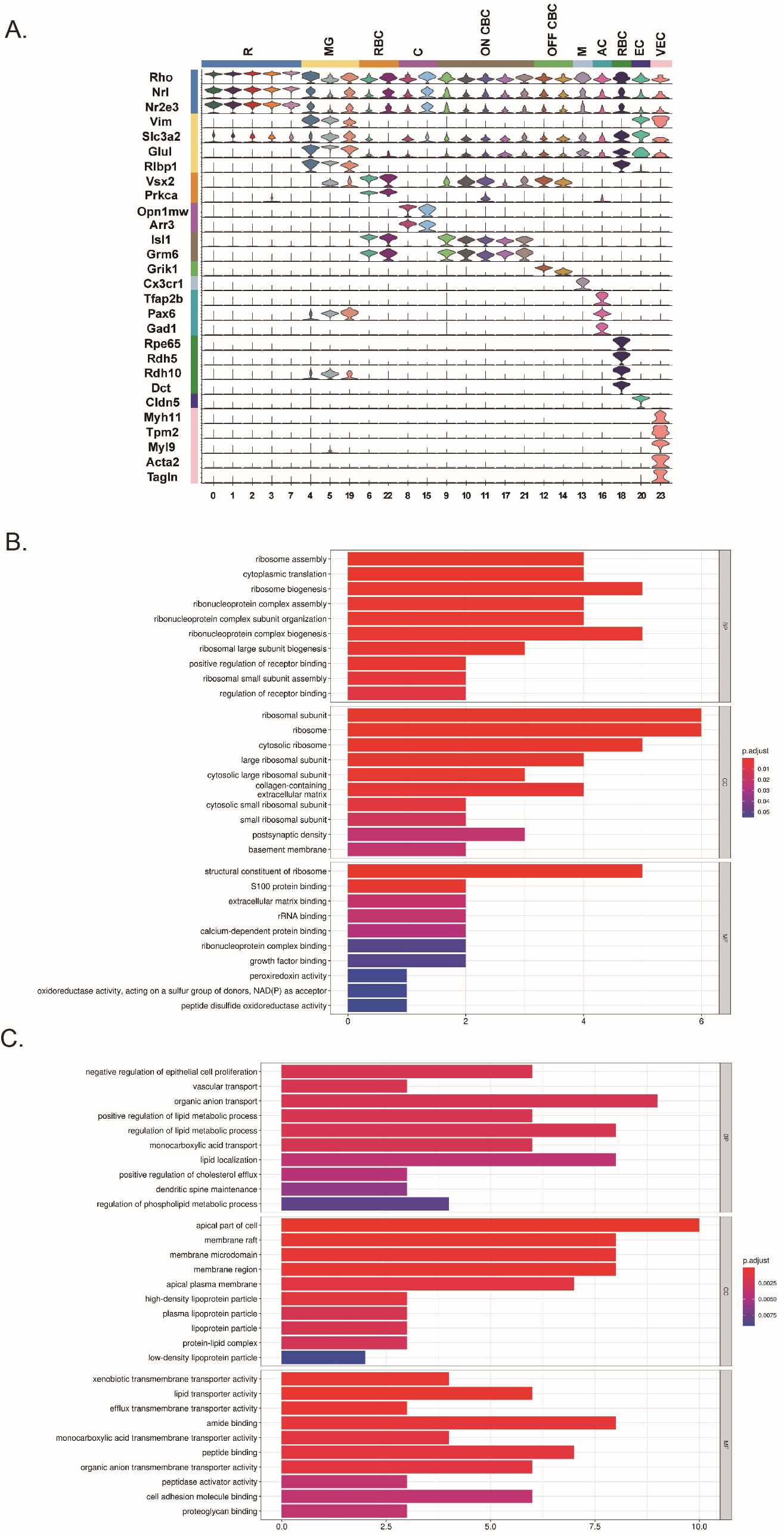
**

**
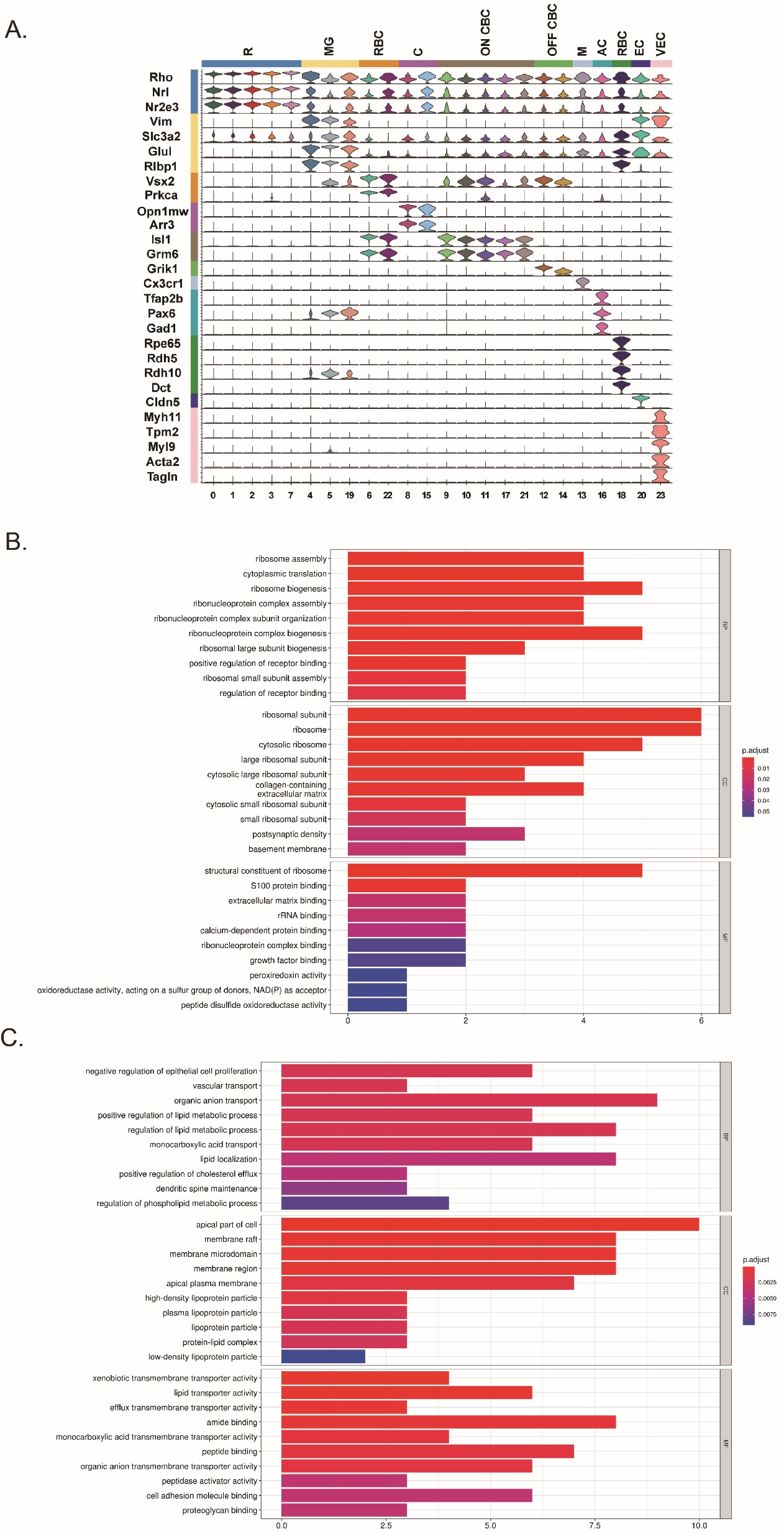
**

**Figure S1.** (A). Cluster-specific expression of marker genes in a violin plot. R: rod photoreceptors; MG: Müller glia; RBC: rod bipolar cells; C: cone photoreceptors; ON CBC： ON cone bipolar cell; OFF CBC：OFF cone bipolar cells； M： microglial； AC: amacrine cells; RPE: retinal pigment epithelial; EC: Vascular endothelial cells; VEC: Vascular smooth muscle cell. (B and C) The downregulated (B) and upregulated (C) gene in VECs GO terms in molecular function (MF), biological process (BP) and cellular component (CC) categories. The color scheme is based on P value from 0.01 (red) to 0.05 (purple).


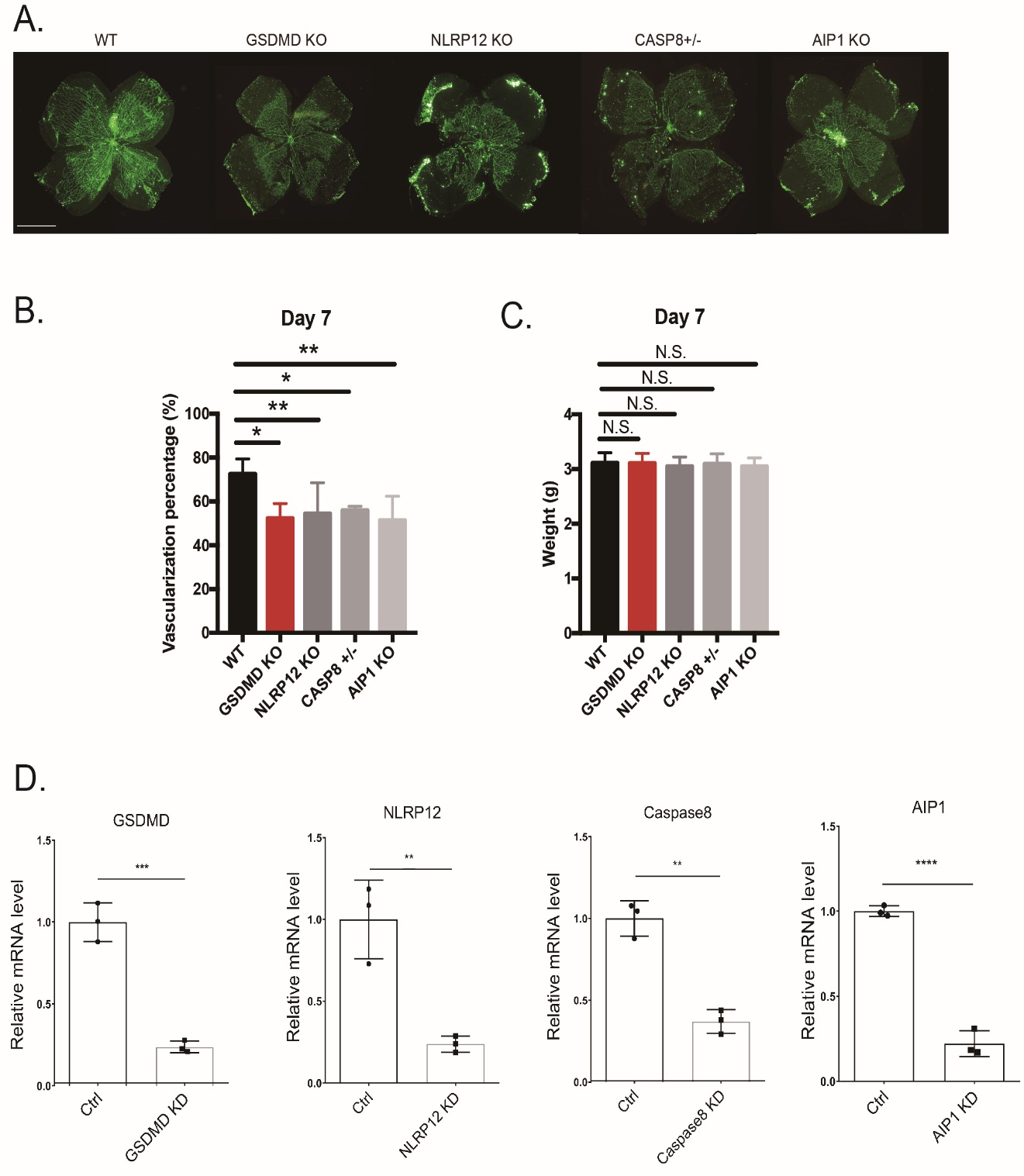


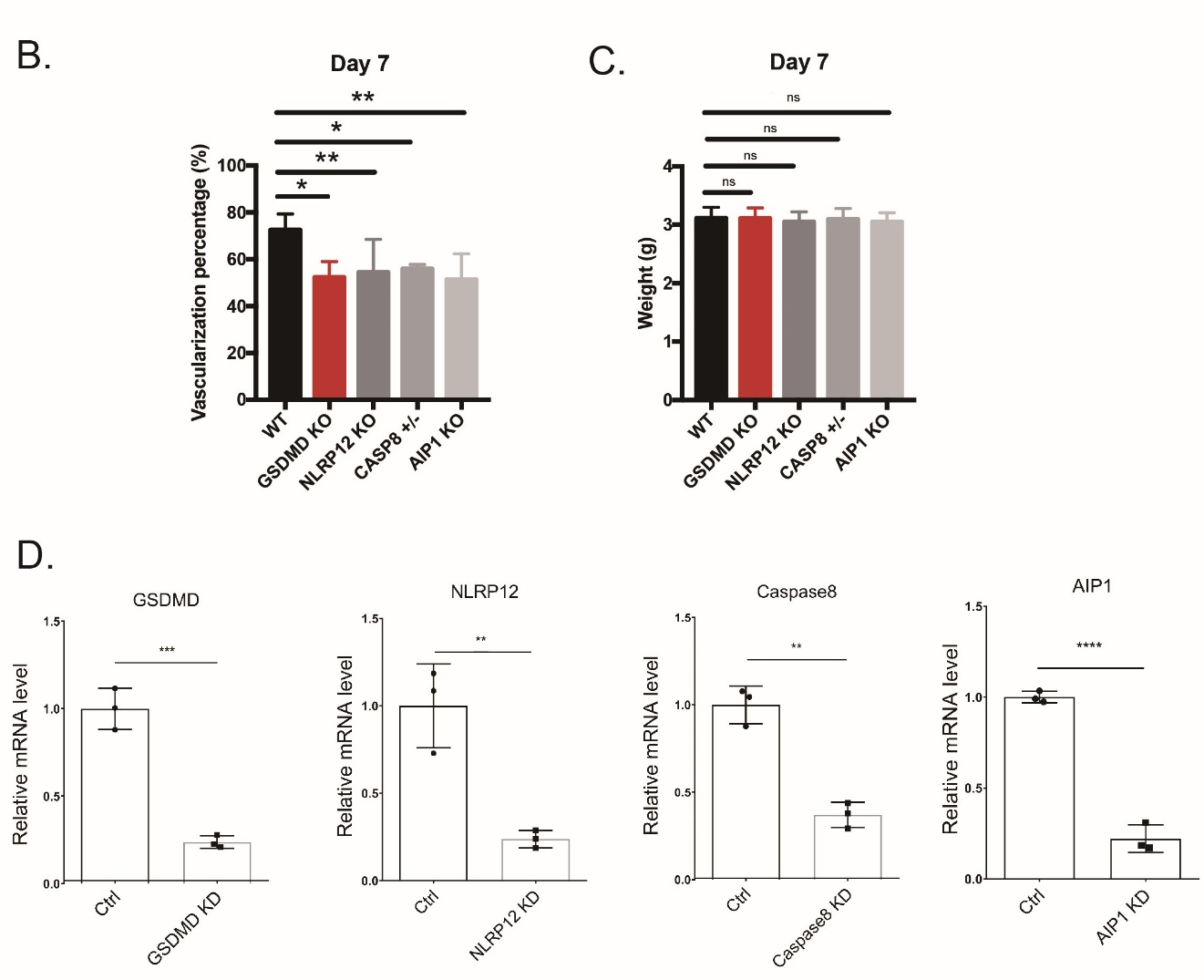


**Figure S2. GSDMD/ NLRP12/ CASP8/ AIP1 gene deletion delayed vascular development.** (A and B) Representative images showing retinal vasculature in different postnatal 7-day mouse strains (n = 6 mice/group). Scale bar: 1 mm. The vascularization area was calculated and analyzed using GraphPad Prism software (B). (C) Body weights of postnatal 7-day mice in different groups were quantitively analyzed by GraphPad Prism software (n = 8 mice/group). (D). The human AIP1, CASP8, NLRP12, and GSDMD siRNAs were used and showed good knockout efficiency (n = 3 mice/group). Data are presented as the mean ± SD. *P < 0.05, **P < 0.01, ns, not significant. Statistical analyses were performed using GraphPad Prism software, with one-way ANOVA followed by Dunnett’s post hoc tests and the independent-sample t-test.

**Reference**

[1] H.L. Goel, A.M. Mercurio, VEGF targets the tumour cell, Nat Rev Cancer, 13 (2013) 871-882.

[2] R.F. Gariano, T.W. Gardner, Retinal angiogenesis in development and disease, Nature, 438 (2005) 960-966.

[3] Y. Sun, Z. Lin, C.H. Liu, Y. Gong, R. Liegl, T.W. Fredrick, S.S. Meng, S.B. Burnim, Z. Wang, J.D. Akula, W.T. Pu, J. Chen, L.E.H. Smith, Inflammatory signals from photoreceptor modulate pathological retinal angiogenesis via c-Fos, J Exp Med, 214 (2017) 1753-1767.

[4] Y. Wooff, S.M. Man, R. Aggio-Bruce, R. Natoli, N. Fernando, IL-1 Family Members Mediate Cell Death, Inflammation and Angiogenesis in Retinal Degenerative Diseases, Front Immunol, 10 (2019) 1618.

[5] W. Chi, F. Li, H. Chen, Y. Wang, Y. Zhu, X. Yang, J. Zhu, F. Wu, H. Ouyang, J. Ge, R.N. Weinreb, K. Zhang, Y. Zhuo, Caspase-8 promotes NLRP1/NLRP3 inflammasome activation and IL-1beta production in acute glaucoma, Proc Natl Acad Sci U S A, 111 (2014) 11181-11186.

[6] A. Kruger-Genge, A. Blocki, R.P. Franke, F. Jung, Vascular Endothelial Cell Biology: An Update, Int J Mol Sci, 20 (2019).

[7] B. Bai, Y. Yang, Q. Wang, M. Li, C. Tian, Y. Liu, L.H.H. Aung, P.F. Li, T. Yu, X.M. Chu, NLRP3 inflammasome in endothelial dysfunction, Cell Death Dis, 11 (2020) 776.

[8] M. Mussbacher, M. Salzmann, C. Brostjan, B. Hoesel, C. Schoergenhofer, H. Datler, P. Hohensinner, J. Basilio, P. Petzelbauer, A. Assinger, J.A. Schmid, Cell Type-Specific Roles of NF-kappaB Linking Inflammation and Thrombosis, Front Immunol, 10 (2019) 85.

[9] A.S. Bharadwaj, B. Appukuttan, P.A. Wilmarth, Y. Pan, A.J. Stempel, T.J. Chipps, E.E. Benedetti, D.O. Zamora, D. Choi, L.L. David, J.R. Smith, Role of the retinal vascular endothelial cell in ocular disease, Prog Retin Eye Res, 32 (2013) 102-180.

[10] H.G. Augustin, G.Y. Koh, Organotypic vasculature: From descriptive heterogeneity to functional pathophysiology, Science, 357 (2017).

[11] N. Lal, K. Puri, B. Rodrigues, Vascular Endothelial Growth Factor B and Its Signaling, Front Cardiovasc Med, 5 (2018) 39.

[12] J.M. Boucher, R.P. Clark, D.C. Chong, K.M. Citrin, L.A. Wylie, V.L. Bautch, Dynamic alterations in decoy VEGF receptor-1 stability regulate angiogenesis, Nat Commun, 8 (2017) 15699.

[13] M.R. Robciuc, R. Kivela, I.M. Williams, J.F. de Boer, T.H. van Dijk, H. Elamaa, F. Tigistu-Sahle, D. Molotkov, V.M. Leppanen, R. Kakela, L. Eklund, D.H. Wasserman, A.K. Groen, K. Alitalo, VEGFB/VEGFR1-Induced Expansion of Adipose Vasculature Counteracts Obesity and Related Metabolic Complications, Cell Metab, 23 (2016) 712-724.

[14] H. Zhang, Y. He, S. Dai, Z. Xu, Y. Luo, T. Wan, D. Luo, D. Jones, S. Tang, H. Chen, W.C. Sessa, W. Min, AIP1 functions as an endogenous inhibitor of VEGFR2-mediated signaling and inflammatory angiogenesis in mice, J Clin Invest, 118 (2008) 3904-3916.

[15] Y.L. Tsai, K.F. Hua, A. Chen, C.W. Wei, W.S. Chen, C.Y. Wu, C.L. Chu, Y.L. Yu, C.W. Lo, S.M. Ka, NLRP3 inflammasome: Pathogenic role and potential therapeutic target for IgA nephropathy, Sci Rep, 7 (2017) 41123.

[16] M. Zhang, C. Xu, H.Z. Wang, Y.N. Peng, H.O. Li, Y.J. Zhou, S. Liu, F. Wang, L. Liu, Y. Chang, Q. Zhao, J. Liu, Soft fibrin matrix downregulates DAB2IP to promote Nanog-dependent growth of colon tumor-repopulating cells, Cell Death Dis, 10 (2019) 151.

[17] M.Y. Zhang, Y.X. Jiang, Y.C. Yang, J.Y. Liu, C. Huo, X.L. Ji, Y.Q. Qu, Cigarette smoke extract induces pyroptosis in human bronchial epithelial cells through the ROS/NLRP3/caspase-1 pathway, Life Sci, 269 (2021) 119090.

[18] P. Ben-Av, L.J. Crofford, R.L. Wilder, T. Hla, Induction of vascular endothelial growth factor expression in synovial fibroblasts by prostaglandin E and interleukin-1: a potential mechanism for inflammatory angiogenesis, FEBS Lett, 372 (1995) 83-87.

[19] B. El Awad, B. Kreft, E.M. Wolber, T. Hellwig-Burgel, E. Metzen, J. Fandrey, W. Jelkmann, Hypoxia and interleukin-1beta stimulate vascular endothelial growth factor production in human proximal tubular cells, Kidney Int, 58 (2000) 43-50.

[20] Y.D. Jung, W. Liu, N. Reinmuth, S.A. Ahmad, F. Fan, G.E. Gallick, L.M. Ellis, Vascular endothelial growth factor is upregulated by interleukin-1 beta in human vascular smooth muscle cells via the P38 mitogen-activated protein kinase pathway, Angiogenesis, 4 (2001) 155-162.

[21] E. Mulvihill, L. Sborgi, S.A. Mari, M. Pfreundschuh, S. Hiller, D.J. Muller, Mechanism of membrane pore formation by human gasdermin-D, EMBO J, 37 (2018).

[22] L. Celkova, S.L. Doyle, M. Campbell, NLRP3 Inflammasome and Pathobiology in AMD, J Clin Med, 4 (2015) 172-192.

[23] J.R. Lukens, P. Gurung, P.J. Shaw, M.J. Barr, M.H. Zaki, S.A. Brown, P. Vogel, H. Chi, T.D. Kanneganti, The NLRP12 Sensor Negatively Regulates Autoinflammatory Disease by Modulating Interleukin-4 Production in T Cells, Immunity, 42 (2015) 654-664.

[24] S.N. Udden, Y.T. Kwak, V. Godfrey, M.A.W. Khan, S. Khan, N. Loof, L. Peng, H. Zhu, H. Zaki, NLRP12 suppresses hepatocellular carcinoma via downregulation of cJun N-terminal kinase activation in the hepatocyte, Elife, 8 (2019).

[25] G.I. Vladimer, D. Weng, S.W. Paquette, S.K. Vanaja, V.A. Rathinam, M.H. Aune, J.E. Conlon, J.J. Burbage, M.K. Proulx, Q. Liu, G. Reed, J.C. Mecsas, Y. Iwakura, J. Bertin, J.D. Goguen, K.A. Fitzgerald, E. Lien, The NLRP12 inflammasome recognizes Yersinia pestis, Immunity, 37 (2012) 96-107.

[26] Q. Xu, T. Qaum, A.P. Adamis, Sensitive blood-retinal barrier breakdown quantitation using Evans blue, Invest Ophthalmol Vis Sci, 42 (2001) 789-794.

[27] S. Tual-Chalot, K.R. Allinson, M. Fruttiger, H.M. Arthur, Whole mount immunofluorescent staining of the neonatal mouse retina to investigate angiogenesis in vivo, J Vis Exp, (2013) e50546.

[28] C.H. Alves, R. Fernandes, A.R. Santiago, A.F. Ambrosio, Microglia Contribution to the Regulation of the Retinal and Choroidal Vasculature in Age-Related Macular Degeneration, Cells, 9 (2020).

[29] J.V. Forrester, L. Kuffova, M. Delibegovic, The Role of Inflammation in Diabetic Retinopathy, Front Immunol, 11 (2020) 583687.

[30] J. Zhang, H.J. Zhou, W. Ji, W. Min, AIP1-mediated stress signaling in atherosclerosis and arteriosclerosis, Curr Atheroscler Rep, 17 (2015) 503.

[31] Q. Huang, L. Qin, S. Dai, H. Zhang, S. Pasula, H. Zhou, H. Chen, W. Min, AIP1 suppresses atherosclerosis by limiting hyperlipidemia-induced inflammation and vascular endothelial dysfunction, Arterioscler Thromb Vasc Biol, 33 (2013) 795-804.

[32] T. Wan, T. Liu, H. Zhang, S. Tang, W. Min, AIP1 functions as Arf6-GAP to negatively regulate TLR4 signaling, J Biol Chem, 285 (2010) 3750-3757.

[33] H. Chen, Y. Deng, X. Gan, Y. Li, W. Huang, L. Lu, L. Wei, L. Su, J. Luo, B. Zou, Y. Hong, Y. Cao, Y. Liu, W. Chi, NLRP12 collaborates with NLRP3 and NLRC4 to promote pyroptosis inducing ganglion cell death of acute glaucoma, Mol Neurodegener, 15 (2020) 26.

[34] H. Chen, X. Gan, Y. Li, J. Gu, Y. Liu, Y. Deng, X. Wang, Y. Hong, Y. Hu, L. Su, W. Chi, NLRP12- and NLRC4-mediated corneal epithelial pyroptosis is driven by GSDMD cleavage accompanied by IL-33 processing in dry eye, Ocul Surf, 18 (2020) 783-794.

[35] S.S. Oladipupo, C. Smith, A. Santeford, C. Park, A. Sene, L.A. Wiley, P. Osei-Owusu, J. Hsu, N. Zapata, F. Liu, R. Nakamura, K.J. Lavine, K.J. Blumer, K. Choi, R.S. Apte, D.M. Ornitz, Endothelial cell FGF signaling is required for injury response but not for vascular homeostasis, Proc Natl Acad Sci U S A, 111 (2014) 13379-13384.

[36] S. Schoors, U. Bruning, R. Missiaen, K.C. Queiroz, G. Borgers, I. Elia, A. Zecchin, A.R. Cantelmo, S. Christen, J. Goveia, W. Heggermont, L. Godde, S. Vinckier, P.P. Van Veldhoven, G. Eelen, L. Schoonjans, H. Gerhardt, M. Dewerchin, M. Baes, K. De Bock, B. Ghesquiere, S.Y. Lunt, S.M. Fendt, P. Carmeliet, Fatty acid carbon is essential for dNTP synthesis in endothelial cells, Nature, 520 (2015) 192-197.

[37] X. Wu, H. Zhang, W. Qi, Y. Zhang, J. Li, Z. Li, Y. Lin, X. Bai, X. Liu, X. Chen, H. Yang, C. Xu, Y. Zhang, B. Yang, Nicotine promotes atherosclerosis via ROS-NLRP3-mediated endothelial cell pyroptosis, Cell Death Dis, 9 (2018) 171.

[38] Y. Zhang, X. Liu, X. Bai, Y. Lin, Z. Li, J. Fu, M. Li, T. Zhao, H. Yang, R. Xu, J. Li, J. Ju, B. Cai, C. Xu, B. Yang, Melatonin prevents endothelial cell pyroptosis via regulation of long noncoding RNA MEG3/miR-223/NLRP3 axis, J Pineal Res, 64 (2018).

[39] T.A. Ferguson, R.S. Apte, Angiogenesis in eye disease: immunity gained or immunity lost?, Semin Immunopathol, 30 (2008) 111-119.

[40] S. Sivaprasad, A.T. Prevost, J.C. Vasconcelos, A. Riddell, C. Murphy, J. Kelly, J. Bainbridge, R. Tudor-Edwards, D. Hopkins, P. Hykin, C.S. Group, Clinical efficacy of intravitreal aflibercept versus panretinal photocoagulation for best corrected visual acuity in patients with proliferative diabetic retinopathy at 52 weeks (CLARITY): a multicentre, single-blinded, randomised, controlled, phase 2b, non-inferiority trial, Lancet, 389 (2017) 2193-2203.

[41] S.B. Bressler, D. Liu, A.R. Glassman, B.A. Blodi, A.A. Castellarin, L.M. Jampol, P.L. Kaufman, M. Melia, H. Singh, J.A. Wells, N. Diabetic Retinopathy Clinical Research, Change in Diabetic Retinopathy Through 2 Years: Secondary Analysis of a Randomized Clinical Trial Comparing Aflibercept, Bevacizumab, and Ranibizumab, JAMA Ophthalmol, 135 (2017) 558-568.

[42] D.K. Wallace, R.T. Kraker, S.F. Freedman, E.R. Crouch, A.K. Hutchinson, A.R. Bhatt, D.L. Rogers, M.B. Yang, K.M. Haider, D.K. VanderVeen, R.M. Siatkowski, T.W. Dean, R.W. Beck, M.X. Repka, L.E. Smith, W.V. Good, M.E. Hartnett, L. Kong, J.M. Holmes, G. Pediatric Eye Disease Investigator, Assessment of Lower Doses of Intravitreous Bevacizumab for Retinopathy of Prematurity: A Phase 1 Dosing Study, JAMA Ophthalmol, 135 (2017) 654-656.

[43] C.K. Domigan, C.M. Warren, V. Antanesian, K. Happel, S. Ziyad, S. Lee, A. Krall, L. Duan, A.X. Torres-Collado, L.W. Castellani, D. Elashoff, H.R. Christofk, A.M. van der Bliek, M. Potente, M.L. Iruela-Arispe, Autocrine VEGF maintains endothelial survival through regulation of metabolism and autophagy, J Cell Sci, 128 (2015) 2236-2248.

[44] J.R. Sierra, S. Corso, L. Caione, V. Cepero, P. Conrotto, A. Cignetti, W. Piacibello, A. Kumanogoh, H. Kikutani, P.M. Comoglio, L. Tamagnone, S. Giordano, Tumor angiogenesis and progression are enhanced by Sema4D produced by tumor-associated macrophages, J Exp Med, 205 (2008) 1673-1685.

[45] L.M. DeBusk, K. Boelte, Y. Min, P.C. Lin, Heterozygous deficiency of delta-catenin impairs pathological angiogenesis, J Exp Med, 207 (2010) 77-84.

[46] H.J. Zhou, X. Chen, Q. Huang, R. Liu, H. Zhang, Y. Wang, Y. Jin, X. Liang, L. Lu, Z. Xu, W. Min, AIP1 mediates vascular endothelial cell growth factor receptor-3-dependent angiogenic and lymphangiogenic responses, Arterioscler Thromb Vasc Biol, 34 (2014) 603-615.

[47] H. Chen, Y. Li, J. Gu, L. Yin, F. Bian, L. Su, Y. Hong, Y. Deng, W. Chi, TLR4-MyD88 pathway promotes the imbalanced activation of NLRP3/NLRP6 via caspase-8 stimulation after alkali burn injury, Exp Eye Res, 176 (2018) 59-68.

[48] Q. Li, X. Hua, L. Li, X. Zhou, Y. Tian, Y. Deng, M. Zhang, X. Yuan, W. Chi, AIP1 suppresses neovascularization by inhibiting the NOX4-induced NLRP3/NLRP6 imbalance in a murine corneal alkali burn model, Cell Commun Signal, 20 (2022) 59.

[49] C. Wei, W. Jiang, R. Wang, H. Zhong, H. He, X. Gao, S. Zhong, F. Yu, Q. Guo, L. Zhang, L.D.J. Schiffelers, B. Zhou, M. Trepel, F.I. Schmidt, M. Luo, F. Shao, Brain endothelial GSDMD activation mediates inflammatory BBB breakdown, Nature, 629 (2024) 893-900.

[50] M. Shibuya, Structure and dual function of vascular endothelial growth factor receptor-1 (Flt-1), Int J Biochem Cell Biol, 33 (2001) 409-420.

[51] S.S. Chaurasia, R.R. Lim, B.H. Parikh, Y.S. Wey, B.B. Tun, T.Y. Wong, C.D. Luu, R. Agrawal, A. Ghosh, A. Mortellaro, E. Rackoczy, R.R. Mohan, V.A. Barathi, The NLRP3 Inflammasome May Contribute to Pathologic Neovascularization in the Advanced Stages of Diabetic Retinopathy, Sci Rep, 8 (2018) 2847.

[52] N. Tisch, A. Freire-Valls, R. Yerbes, I. Paredes, S. La Porta, X. Wang, R. Martin-Perez, L. Castro, W.W. Wong, L. Coultas, B. Strilic, H.J. Grone, T. Hielscher, C. Mogler, R.H. Adams, P. Heiduschka, L. Claesson-Welsh, M. Mazzone, A. Lopez-Rivas, T. Schmidt, H.G. Augustin, C. Ruiz de Almodovar, Caspase-8 modulates physiological and pathological angiogenesis during retina development, J Clin Invest, 129 (2019) 5092-5107.

[53] L. Todd, I. Palazzo, L. Suarez, X. Liu, L. Volkov, T.V. Hoang, W.A. Campbell, S. Blackshaw, N. Quan, A.J. Fischer, Reactive microglia and IL1beta/IL-1R1-signaling mediate neuroprotection in excitotoxin-damaged mouse retina, J Neuroinflammation, 16 (2019) 118.

[54] K. Shekhar, S.W. Lapan, I.E. Whitney, N.M. Tran, E.Z. Macosko, M. Kowalczyk, X. Adiconis, J.Z. Levin, J. Nemesh, M. Goldman, S.A. McCarroll, C.L. Cepko, A. Regev, J.R. Sanes, Comprehensive Classification of Retinal Bipolar Neurons by Single-Cell Transcriptomics, Cell, 166 (2016) 1308-1323 e1330.

[55] B.T. Kalish, L. Cheadle, S. Hrvatin, M.A. Nagy, S. Rivera, M. Crow, J. Gillis, R. Kirchner, M.E. Greenberg, Single-cell transcriptomics of the developing lateral geniculate nucleus reveals insights into circuit assembly and refinement, Proc Natl Acad Sci U S A, 115 (2018) E1051-E1060.

[56] W. Samuel, C. Jaworski, O.A. Postnikova, R.K. Kutty, T. Duncan, L.X. Tan, E. Poliakov, A. Lakkaraju, T.M. Redmond, Appropriately differentiated ARPE-19 cells regain phenotype and gene expression profiles similar to those of native RPE cells, Mol Vis, 23 (2017) 60-89.

[57] A.S. Kalluri, S.K. Vellarikkal, E.R. Edelman, L. Nguyen, A. Subramanian, P.T. Ellinor, A. Regev, S. Kathiresan, R.M. Gupta, Single-Cell Analysis of the Normal Mouse Aorta Reveals Functionally Distinct Endothelial Cell Populations, Circulation, 140 (2019) 147-163.
